# Ultralong Hydroxyapatite Nanowires: Promising Flexible Building Blocks for Constructing High-Performance Biomimetic Materials—A Review

**DOI:** 10.3390/molecules31010142

**Published:** 2026-01-01

**Authors:** Han-Ping Yu, Ying-Jie Zhu

**Affiliations:** 1 Shanghai Institute of Ceramics, Chinese Academy of Sciences, Shanghai 200050, China; yuhanping@mail.sic.ac.cn; 2 Center of Materials Science and Optoelectronics Engineering, University of Chinese Academy of Sciences, Beijing 100049, China

**Keywords:** flexible nanofiber, biomimetic materials, hydroxyapatite nanowires, biomaterial engineering, nanostructured materials

## Abstract

Traditional hydroxyapatite materials are inherently stiff and brittle, limiting their applications. Flexible ultralong hydroxyapatite nanowires, characterized by nano-scale diameters and micrometer-scale lengths, offer a promising alternative as one-dimensional flexible building blocks for constructing high-performance biomimetic materials. Nature has evolved a variety of high-performance materials with hierarchically ordered structures assembled from nano-scale building blocks, which provide valuable insights into the design and ordered assembly of flexible nanofibers for building high-performance biomimetic materials. Currently, how to distill the structural design principles of natural materials to engineer flexible nanofibers into advanced high-performance biomimetic materials with excellent properties and multifunctions remains a frontier scientific challenge. In 2014, the authors’ research group reported for the first time the calcium oleate precursor solvothermal method for the synthesis of flexible ultralong hydroxyapatite nanowires and their applications. Since then, many soft functional materials and high-performance biomimetic materials have been designed and prepared using flexible ultralong hydroxyapatite nanowires, and their applications in various fields have been explored. These studies demonstrate the successful assembly of flexible ultralong hydroxyapatite nanowires into hierarchical biomimetic structures inspired by natural materials such as enamel, nacre, and bone, which exhibit enhanced mechanical properties, including improved strength, toughness, and flexibility, alongside multifunctional capabilities like thermal insulation and biomedical compatibility. These findings suggest that flexible ultralong hydroxyapatite nanowires provide a versatile platform for designing and constructing advanced biomimetic materials with promising applications in various fields. This review article aims to briefly review recent advances in this exciting and rapidly evolving research field. The synthetic methods, assembly strategies, properties, and applications of flexible ultralong hydroxyapatite nanowires and their derivative biomimetic materials are discussed, enlightening their structural design principles and potential applications. Finally, we propose future research directions and future perspectives in this exciting frontier research field.

## 1. Introduction

Through billions of years of evolution, nature has developed a wide variety of high-performance structural and functional biomaterials that fulfill essential mechanical and biological roles in organisms [1,2]. Remarkably, these natural biomaterials are composed of hierarchically ordered structures, precisely assembled from nano-scale building blocks [3]. Such sophisticated natural architectures have inspired the design and fabrication of high-performance biomimetic materials, guided by the structure–property relationships observed in nature [3,4,5,6,7]. These bioinspired materials have demonstrated strong competitiveness in diverse fields, including mechanical engineering, advanced biomaterials, safety protection, energy storage, and aerospace engineering [8,9,10,11,12,13]. Furthermore, biomimicry offers valuable strategies for addressing challenges in materials science [3,14]. To meet survival needs, organisms have evolved unique biomaterials with optimized structures and multifunctionality, often combining properties that are typically mutually exclusive. Consequently, biomimetic structural designs have enabled the creation of materials that combine high strength with high toughness, lightweight with high strength, and superior mechanical properties with high bioactivity—combinations that are difficult to achieve in traditional materials [3]. Thus, biomimetic materials play a crucial role in advancing high-performance artificial materials and expanding their applications in various fields.

The composition, morphology, size of building blocks, and microstructure are critical factors influencing the physicochemical properties and macroscopic performance of materials. For example, ceramic materials are typically monoliths composed of numerous stacked crystal grains [15,16]. The strong ionic or covalent bonds within these grains impart ceramics with high stiffness, strength, and temperature resistance; however, these bonds weaken rapidly as interatomic distances increase, leading to undesirable brittleness. Achieving strong and tough ceramic materials has long been a challenge in practical applications. Recently, the development of flexible inorganic nanofibers has opened new avenues for constructing high-performance biomimetic materials with promising applications. Notable examples include ultralong hydroxyapatite (HAP) nanowires [17], TiO_2_ nanofibers [18], ZrO_2_–SiO_2_ nanofibers [19], SiO_2_ nanofibers [20], SiC–SiO_x_ nanowires [21], and BaSO_4_ nanorod-assembled fibers [22]. These inorganic nanofibers typically exhibit high aspect ratios and can bend naturally at the micro-scale [23]. Materials assembled from high-aspect-ratio inorganic nanofibers display greater flexibility and toughness than their monolithic counterparts. In comparison to organic fiber-based structural materials, high-aspect-ratio inorganic nanofiber-based materials not only offer high flexibility but also possess superior chemical stability, modulus, aging resistance, and thermal stability, making them indispensable in various applications [24,25]. Additionally, high-aspect-ratio flexible inorganic nanofibers can simultaneously enhance both the strength and toughness of the resulting materials.

Many traditional materials suffer from poor mechanical properties and limited functionality, which severely restricts their versatility and reliability across a wide range of applications. Therefore, there is an urgent need to develop new approaches for engineering high-aspect-ratio flexible inorganic nanofibers into high-performance advanced materials with superior mechanical properties and multifunctionality. One effective strategy is to assemble flexible inorganic nanofibers into biomimetic architectures. Many examples of biomineralized fibrous materials exist in nature, such as enamel, bone, and nacre, which are composed of one-dimensional (1-D) nano-scale inorganic or mineralized building blocks. A key feature of these biomineralized fibrous materials is their ability to overcome the brittleness of inorganic building blocks by transferring the superior properties of nano-scale components to the macroscopic scale through hierarchically assembled, preferentially oriented structures [3]. Such structural designs are highly effective in significantly enhancing the mechanical performance of materials.

Despite extensive research and rapid progress in biomimetic materials, few have been developed based on high-aspect-ratio flexible inorganic nanofibers. Given the unique combination of strength and flexibility offered by these nanofibers, as well as the structural designs found in natural materials, integrating flexible inorganic nanofibers into biomimetic architectures is expected to enhance the properties and expand the applications of high-performance artificial materials. However, a key scientific challenge remains: how to extract and apply the structural design principles from natural materials to engineer flexible inorganic nanofibers into advanced high-performance biomimetic materials.

Ultralong hydroxyapatite (HAP) nanowires are defined as hydroxyapatite nanowires with diameters less than 100 nm, lengths greater than 100 μm, and aspect ratios exceeding 1000 [26]. In 2014, the authors’ research group first reported the calcium oleate precursor solvothermal method for synthesizing high-aspect-ratio flexible ultralong HAP nanowires and explored their applications [17]. Since then, many soft functional materials and high-performance biomimetic materials have been developed using flexible ultralong HAP nanowires, with applications spanning biomedicine, specialty paper, fire resistance, high-temperature tolerance, energy storage, environmental protection, and flexible fire-resistant electronics [26,27]. This article reviews recent advances in superior biomimetic materials based on flexible ultralong HAP nanowires. It discusses the synthetic methods, assembly strategies, properties, and applications of flexible ultralong HAP nanowires and their derivative biomimetic materials, highlighting their structural design principles and potential applications. Finally, we discuss current research trends and challenges, and propose future research directions in this exciting frontier field.

## 2. High-Aspect-Ratio Flexible Ultralong HAP Nanowires: Towards New Functionalities and Superior Mechanical Properties of Biomimetic Materials

HAP is the most thermodynamically stable phase in the calcium phosphate family [28]. Its chemical composition closely resembles that of the mineral component in natural bone and teeth, and HAP biomaterials exhibit excellent biocompatibility, bioactivity, and superior osteoconductive properties [29,30]. Consequently, HAP biomaterials are widely used in biomedical fields such as bone defect repair, dental restoration, wound healing, bio-imaging, and drug delivery [31,32,33]. In living organisms, HAP serves as the primary inorganic component that provides strength and rigidity to biomineralized tissues, reinforcing their microstructure and enhancing mechanical properties. Additionally, HAP materials possess other advantageous properties [34], including a high melting point (about 1650 °C), high physicochemical stability, resistance to aging and high temperatures, non-flammability, and high whiteness. However, similar to most ceramic materials, traditional HAP materials are highly brittle, which significantly limits their practical applications.

**Figure 1:**
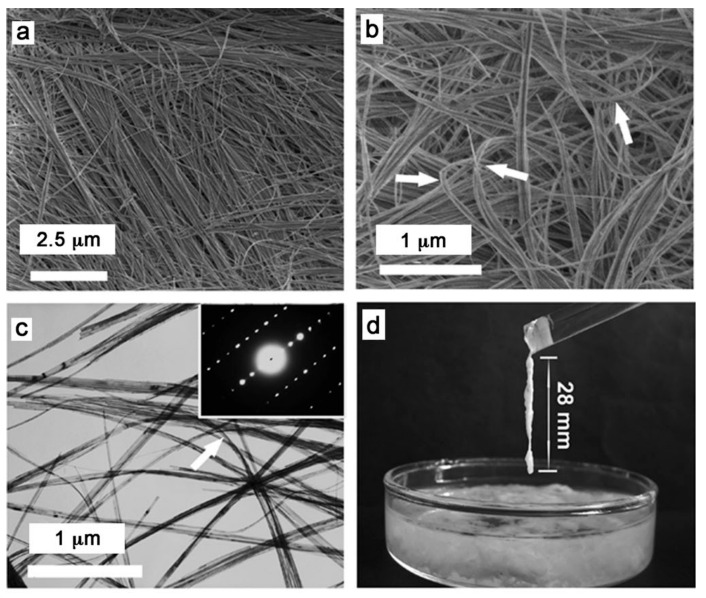
(**a**–**c**) Flexible ultralong HAP nanowires synthesized by the calcium oleate precursor solvothermal method: (**a**,**b**) scanning electron microscopy (SEM) images; (**c**) transmission electron microscopy (TEM) image. (**d**) Digital image of a long macroscopic fiber consisting of ultralong HAP nanowires. The arrows in (**b**,**c**) show the bending of ultralong HAP nanowires. Reproduced with permission from ref. [17]. Copyright 2014, Wiley-VCH.

It is well-known that the morphology of building blocks significantly affects the properties of materials. Traditional HAP materials exhibit high brittleness because they consist of HAP particles, rods, or sheets, all of which have poor flexibility [35,36,37,38]. It is difficult to prepare deformable materials based on these rigid HAP structures as building blocks. To tackle this challenge, the authors’ research group reported for the first time the calcium oleate precursor solvothermal method for the synthesis of a novel kind of highly flexible ultralong HAP nanowires in 2014 (Figure 1) [17]. The as-prepared ultralong HAP nanowires possess diameters of about ten nanometers and lengths up to hundreds of micrometers, with ultrahigh aspect ratios more than 10,000 and high flexibility. The morphological investigations of flexible ultralong HAP nanowires indicate that they can bend naturally and tend to self-assemble into nanowire bundles with larger diameters along the longitudinal direction at the nano-scale. Owing to their unique advantages, ultralong HAP nanowires offer a promising candidate to construct flexible biomimetic materials, which may help address the brittleness and stiffness problems of traditional HAP materials.

Currently, inorganic compound nanofibers reported in the literature are mainly oxide nanofibers, and their aspect ratios and lengths are usually small. In contrast, compared with other kinds of inorganic compound nanofibers, ultralong HAP nanowires show smaller diameters (~10 nm) and much higher aspect ratios (more than 10,000). Furthermore, HAP is the main inorganic component of natural hard tissues such as teeth and bone, thus exhibiting superior biocompatibility, bioactivity, and bio-safety compared with other inorganic compound nanofibers. Therefore, flexible ultralong HAP nanowires provide a versatile platform for designing and constructing high-performance advanced biomimetic materials with promising applications in various fields.

By using flexible ultralong HAP nanowires as the building blocks, various forms of deformable materials, such as twistable orderly nanostructured ropes/ribbons [39,40], flexible fire-resistant paper [17,26,27,41], elastic aerogels [42], deformable scaffolds [43], and bendable hydrogels [44,45], were developed, greatly expanding the applications of HAP materials beyond traditional bio-ceramics to novel multifunctional flexible materials. Although natural HAP-based biomineralized tissues such as bone and enamel show high mechanical performance, they are composed of HAP nanorods or nanosheets as the building blocks with inferior toughness. Ultralong HAP nanowires exhibit better morphology and mechanical properties than natural HAP building blocks. Therefore, assembling ultralong HAP nanowires into biomimetic structural materials may provide a pathway to develop superior biomimetic materials with enhanced properties.

## 3. Synthetic Methods for Highly Flexible Ultralong HAP Nanowires

As the building blocks for the construction of high-performance biomimetic materials, the high-quality and large-scale production of flexible ultralong HAP nanowires is essential. In this section, we outline the research journey in the successful synthesis of flexible ultralong HAP nanowires, covering their formation process, influencing factors, morphological control, optimization of preparation conditions, considerations for large-scale production, and energy-saving strategies (such as shortening synthetic time). These considerations, along with the optimization of synthetic strategies, may provide important references for large-scale production and applications of other flexible inorganic nanofibers.

### 3.1. Calcium Oleate Precursor Solvothermal Method

The flexible ultralong HAP nanowires can be synthesized through the calcium oleate precursor solvothermal method reported by our research group [17]. The calcium oleate precursor is synthesized at room temperature using CaCl_2_, NaOH, and oleic acid in a mixture of ethanol and water. Subsequently, the calcium oleate precursor and its mother reaction system are mixed with the NaH_2_PO_4_ aqueous solution, and the resulting reaction system is solvothermally treated at 160~220 °C for different times (5~48 h). Ethanol is added into the liquid mixture obtained after solvothermal treatment to flocculate ultralong HAP nanowires. The resulting liquid suspension containing flocculated ultralong HAP nanowires is filtrated, and the solid product is washed with ethanol and water several times to obtain flexible ultralong HAP nanowires.

The calcium oleate precursor solvothermal method is applicable to the synthesis of ultralong HAP nanowires using various monohydroxy alcohols as the solvent, for instance, methanol, ethanol, 1-propanol, 2-propanol, 1-butanol, 1-pentanol, and 1-hexanol [46]. Notably, the type of monohydroxy alcohol affects the length of the as-prepared ultralong HAP nanowires. Flexible ultralong HAP nanowires with lengths up to approximately 1 mm can be obtained by this method using methanol as the solvent. Moreover, the type of inorganic phosphate salt also influences the morphology and length of ultralong HAP nanowires. Zhang et al. [47,48] investigated the influence of different phosphate salts, such as NaH_2_PO_4_·2H_2_O, Na_2_HPO_4_·12H_2_O, Na_3_PO_4_·12H_2_O, Na_5_P_3_O_10_, Na_4_P_2_O_7_·10H_2_O, and (NaPO_3_)_6_ on the product prepared by the calcium oleate precursor solvothermal method using a mixed solvent of water and ethanol. They discovered that the flexible ultralong HAP nanowires could be synthesized via the calcium oleate precursor solvothermal method using various phosphates as the phosphorus source, except for (NaPO_3_)_6_. In the case of using (NaPO_3_)_6_ as the phosphorus source, ultralong HAP microtubes instead of ultralong HAP nanowires were obtained under the same reaction conditions [47,48]. In addition, the initial Ca/P molar ratio influences the morphology and selective orientation of ultralong HAP nanowires and HAP nanowires with high aspect ratios formed within an appropriate initial Ca/P molar ratio range [49].

The cost for the preparation of ultralong HAP nanowires can be reduced by the use of cheaper raw materials. For example, the large-scale, green, and low-cost synthesis of ultralong HAP nanowires was achieved using peanut oil [50]. Peanut oil is composed of a high content of oleic acid; thus, it can replace oleic acid to react with Ca^2+^ ions and form calcium oleate precursor. Peanut oil is a healthy cooking oil, and its use in synthesizing ultralong HAP nanowires can avoid pollution and reduce the cost. It is promising for the industry-scale production of ultralong HAP nanowires in the future.

Generally, the calcium oleate precursor solvothermal method enables the synthesis of ultralong HAP nanowires with extremely high aspect ratios (exceeding 10,000) and allows for control over the nanowire morphology and assembly through solvent selection. However, due to the use of organic solvents, its cost, requirements for the reaction equipment (including pressure, corrosion resistance, etc.), and environmental impact cannot be overlooked and must be comprehensively considered in the practical large-scale production of ultralong HAP nanowires.

### 3.2. Calcium Oleate Precursor Hydrothermal Method

Considering the high cost of organic solvents and their environmental impact, the low-cost calcium oleate precursor hydrothermal method using water as the only solvent without any organic solvent was developed by the authors’ research group for the synthesis of ultralong HAP nanowires [51]. In this environmentally friendly method, water-soluble calcium salt is used as the calcium source, water-soluble phosphate salt is used as the phosphorus source, and oleate salt such as sodium oleate is used as oleate source to replace oleic acid. Moreover, the oleate salt can be substituted by other aliphatic acid salts with similar structures, such as sodium stearate and sodium laurate [51]. The calcium oleate precursor hydrothermal method is safer and more economic than the calcium oleate precursor solvothermal method; thus, it is more promising to be scaled up for the production of flexible ultralong HAP nanowires. In the authors’ laboratory, we have successfully achieved the scaled-up production of flexible ultralong HAP nanowires using stainless steel autoclaves with a volume of 10 L and even 100 L by the calcium oleate precursor hydrothermal method [51].

Compared with the calcium oleate precursor solvothermal method using organic solvents, the calcium oleate precursor hydrothermal method using water as the only solvent is safer, has a lower cost, and is more environmentally friendly, which makes it promising for the large-scale production of flexible ultralong HAP nanowires. However, due to the pure water environment of the calcium oleate precursor hydrothermal method, the calcium oleate precursor is obtained through the chemical reaction of a calcium source with oleate salts. The oleate salts are generally poorly soluble in water, and if high concentrations are required, heating is usually needed to promote the dissolution of oleate salts.

### 3.3. Calcium Oleate Precursor Microwave-Assisted Hydrothermal/Solvothermal Method

The calcium oleate precursor hydrothermal/solvothermal method usually requires tens of hours to synthesize flexible ultralong HAP nanowires due to low heating efficiency. In comparison, microwave-assisted heating can rapidly elevate the temperature of the reaction system, and thus it is usually conjunct with the hydrothermal/solvothermal method to enhance the reaction efficiency for material synthesis [52,53,54]. In this case, the synthetic rate of flexible ultralong HAP nanowires can be significantly accelerated by the microwave-assisted hydrothermal/solvothermal method. Yu et al. [55] investigated the applicability of the calcium oleate precursor microwave-assisted hydrothermal/solvothermal method for the rapid synthesis of flexible ultralong HAP nanowires. This method possesses merits of high efficiency, greatly shortened preparation time, low cost, and energy saving. Notably, flexible ultralong HAP nanowires could be rapidly formed within just 20 min, representing a significant reduction in synthetic time by nearly two orders of magnitude compared to the conventional hydrothermal/solvothermal method using the calcium oleate precursor. Moreover, owing to the uniform heating mode of microwave irradiation, the as-prepared flexible ultralong HAP nanowires show relatively uniform morphology and size.

Therefore, the calcium oleate precursor microwave-assisted hydrothermal/solvothermal method shows merits such as high efficiency, greatly shortened preparation time, low cost, and energy saving. This method can prepare relatively uniform flexible ultralong HAP nanowires within 1 h, which is highly efficient compared with the conventional hydrothermal/solvothermal method. However, this method also has some limitations, for example, the application of this method is still limited to the laboratory-scale rapid synthesis of ultralong HAP nanowires, and the large-scale production of ultralong HAP nanowires needs a special large-sized microwave reactor, which is still a major challenge.

### 3.4. Calcium Oleate Precursor Phosphorus-Containing Biomolecule Microwave-Assisted Hydrothermal Method

Nucleation and crystal growth are important to the formation of ultralong HAP nanowires. The conventional methods for the formation of HAP materials are based on the chemical reaction between calcium salt and inorganic phosphate salt in aqueous solution. However, inorganic phosphate salts will rapidly form free phosphate (PO_4_^3–^) ions by ionization in aqueous solution, resulting in the rapid and uncontrollable reaction between calcium ions and phosphate ions. To tackle this problem, it is necessary to control the formation rate of free phosphate ions in aqueous solution. In fact, the phosphate group not only exists in inorganic phosphate salts, but also in the phosphorus-containing biomolecules [56,57,58]. Compared with inorganic phosphate salts, the phosphorus-containing biomolecules as the phosphorus source have several advantages [59], for example, (1) the phosphate groups exist in the biomolecules and will only hydrolyze under specific conditions, avoiding the rapid release of phosphate ions and premature nucleation due to the uncontrolled reaction between calcium ions and phosphate ions; (2) the concentration of phosphate ions released from the phosphorus-containing biomolecules can be well-controlled by the hydrolyzation parameters; (3) it is feasible to control the nucleation rate and crystal growth of HAP according to the hydrolyzation conditions of phosphorus-containing biomolecules; (4) the functional groups within some phosphorus-containing biomolecules are able to control the morphology and structure of HAP nanostructures; (5) some bioactive groups of phosphorus-containing biomolecules can be in situ decorated on the surface of HAP nanostructures and thus enhance their bioactivity; and (6) phosphorus-containing biomolecules are highly biocompatible with high biosafety.

Zhang et al. [60] developed the calcium oleate precursor phosphorus-containing biomolecule microwave-assisted hydrothermal method using biocompatible adenosine 5′-triphosphate (ATP) as a bio-phosphorus source and water as the only solvent for the rapid synthesis of ultralong HAP nanowires. The controllable hydrolysis of ATP biomolecules could avoid the premature formation of calcium phosphate nuclei and uncontrollable crystal growth. Microwave heating could significantly shorten the synthetic time from tens of hours required by the traditional heating to 1 h, thus achieving high efficiency, energy saving, and low cost. The as-prepared ultralong HAP nanowires exhibited high bioactivity and biocompatibility owing to the bioactive adenosine adsorbed on the surface of ultralong HAP nanowires. Ultralong HAP nanowires prepared by this method are promising for various applications in the biomedical fields, such as bone defect repair, skin wound healing, and as a drug nanocarrier.

In another work, Zhang et al. [61] reported the calcium oleate precursor phosphorus-containing biomolecule microwave-assisted hydrothermal method using biogenic creatine phosphate as the bio-phosphorus source for the rapid synthesis of ultralong HAP nanowires. The as-prepared ultrathin ultralong HAP nanowires were several nanometers in diameter, several hundred micrometers in length, and had ultrahigh aspect ratios (>10,000). This method could effectively shorten the synthesis time by about two orders of magnitude compared with the traditional hydrothermal method. In addition, ultrathin ultralong HAP nanowires were decorated on the surface in situ with bioactive creatine and self-assembled into nanowire bundles along their longitudinal direction at the nano-scale.

As discussed above, the nucleation and crystal growth during the synthetic process of flexible ultralong HAP nanowires can be well-controlled by using phosphorus-containing biomolecules as the phosphorus source under microwave heating. The phosphorus-containing biomolecules do not release free PO_4_^3−^ ions at room temperature in aqueous solution, which avoids premature nucleation and uncontrolled crystal growth. In addition, microwave heating can rapidly elevate the temperature of the reaction system to accelerate the chemical reaction and crystal growth, significantly shortening the preparation time [60,61].

The calcium oleate precursor phosphorus-containing biomolecule microwave-assisted hydrothermal method not only enables the rapid synthesis of ultralong HAP nanowires with the relatively uniform morphology and high biocompatibility and bioactivity, but also allows for precise control over the chemical reaction, nucleation, and growth processes through the regulated hydrolysis of phosphorus-containing biomolecules. This approach yields ultrathin ultralong HAP nanowires and enables their in situ surface modification of biocompatible and bioactive constituents, resulting in ultralong HAP nanowires highly sought after for biomedical applications. More importantly, the flexible ultralong HAP nanowires prepared by this method have ultrasmall diameters of about 6 nm, which are smaller than those prepared by the calcium oleate precursor hydrothermal method using conventional inorganic phosphate (usually more than 10 nm). However, this approach suffers from some drawbacks, for example, it is still limited to the laboratory-scale rapid synthesis of ultralong HAP nanowires, and the large-scale production of ultralong HAP nanowires needs a special large-sized microwave reactor, which is still a major challenge. In addition, phosphorus-containing biomolecules are usually expensive, thus, leading to high cost for the synthesis of ultralong HAP nanowires.

## 4. Rational Designs and Construction of Biomimetic Materials Based on Ultralong HAP Nanowires

Owing to the exceptional combination of strength and flexibility in ultralong HAP nanowires, they are an ideal candidate for constructing high-performance biomimetic materials by mimicking the structures of natural materials. There are various kinds of HAP-based materials in organisms, such as enamel, compact bone, cancellous bone, and fish scale. They perform distinct functions by means of specific orderly assembled structures, offering valuable insights into the rational designs for biomimetic materials to achieve high mechanical performance and tailored biological functions. In this section, we illustrate how to rationally design high-performance biomimetic materials based on ultralong HAP nanowires by mimicking the structures of natural biomaterials.

### 4.1. Enamel-Mimetic Multi-Scale Ordered Structure: Significantly Enhanced Mechanical Properties

Enamel, the outermost layer of tooth, is known as the hardest tissue in nature owing to its highly mineralized and organized nanostructured structure. HAP nanofibers are the primitive building blocks of enamel. They are densely arranged in an orderly manner along their longitudinal direction. A remarkable structural feature of enamel is the hierarchical multi-scale ordered structure (from the nano-scale to the micro-scale to the macro-scale) [3,62]. In recent years, there has been increasing attention paid to mimicking the multi-scale ordered structure of enamel [63]. Considering that ultralong HAP nanowires are similar to HAP nanofibers in enamel, both are one-dimensional nanostructures, it is feasible to duplicate the hierarchical ordered structure and excellent mechanical properties of enamel using ultralong HAP nanowires as the building blocks.

Yu et al. [64] developed a bottom-up step-by-step assembly strategy to build the tooth enamel-mimetic structural materials based on ultralong HAP nanowires reinforced with resin, and constructed a multi-scale (from the nano-scale to the micro-scale to the macro-scale) highly ordered HAP nanowire structure. In this work, ultralong HAP nanowires were assembled in sequence into highly ordered HAP nanowire bundles, aligned HAP microfibers, and 3-D highly ordered HAP nanowire bulk, which perfectly imitated the structure of the tooth enamel. The as-prepared enamel-mimetic structural materials with unique multi-scale highly ordered structure exhibited significant enhancement of the macroscopic compressive Young’s modulus (2.80 GPa), which was 3.54 times that of the pure resin and 28 times that of the highly ordered HAP nanowire bulk sample. Owing to the high flexibility of ultralong HAP nanowires, the enamel-mimetic structural materials showed sufficient toughness for material processing, enabling the fabrication of various products with different sizes and shapes. For example, a typical enamel-mimetic structural material with a diameter of about 1 cm and a length of more than 6 cm was prepared, which is the largest enamel-mimetic structural material based on ultralong HAP nanowires reported in the literature. Additionally, in fractured enamel-mimetic structural materials, various fracture behaviors similar to those in natural structural materials were observed, such as nanowire separation, nanowire fracture, crack deflection, and zigzag microcracks. In contrast, only a straight crack path and smooth fracture surface could be observed in the pure resin.

In addition to the hierarchical multi-scale ordered structure, the intergranular structure and constituents between adjacent HAP nanocrystals are also crucial to the mechanical performance and properties of enamel [65,66,67,68]. For example, the stability of grain boundaries in enamel is enhanced by Mg- and Fe-stabilized amorphous ingredients and the elemental gradient structure between HAP nanofibers, which improves the mechanical properties of enamel and increases the resistance to acid erosion. Inspired by this guideline, Zhao et al. [69] reported an enamel-mimetic hierarchical structure at multiple scales by the self-assembly of amorphous ZrO_2_ intergranular phase-coated hydroxyapatite nanowires intertwined with polyvinyl alcohol, which showed high stiffness, hardness, strength, viscoelasticity, and toughness. During the preparation process, dual-directional freezing of dispersions containing amorphous ZrO_2_-coated hydroxyapatite nanowires with polyvinyl alcohol was adopted, and a polydimethylsiloxane wedge formed a bi-directional temperature gradient, driving the ice crystal growth in perpendicular and parallel directions. The perpendicular growth of the ice crystals forced the amorphous ZrO_2_-coated hydroxyapatite nanowires and polyvinyl alcohol to occupy the gaps between ice lamellae, and the parallel growth forced them to form a parallel orientation, and after mechanical compression, the dense artificial tooth enamel was obtained. The amorphous ZrO_2_ intergranular phase could act as a buffer layer to facilitate stress transfer and enhance the inorganic–organic interfacial connection, thus contributing to the excellent mechanical performance.

In another research work, Chen et al. [70] prepared ultralong HAP nanowires with a layer of an Fe^3+^-rich amorphous intergranular phase on the outer surface layer by ion exchange between Fe^3+^ and Ca^2+^ in the HAP lattice. By bi-directional freezing of Fe-HAP nanowires in the presence of polyvinyl alcohol, rodent enamel-mimetic nanocomposite was obtained, showing a hardness of 1.66 ± 0.23 GPa and a Young’s modulus of 48.8 ± 6.7 GPa in the longitudinal direction, and a hardness of 1.38 ± 0.16 GPa and a Young’s modulus of 40.2 ± 3.0 GPa in the transversal direction. The experimental results indicated that the mechanical properties of the rodent enamel-mimetic nanocomposite were greatly enhanced compared with the sample without the Fe^3+^-rich amorphous intergranular phase.

### 4.2. Nacre-Mimetic “Brick-and-Mortar” Structure: Towards High Toughness Biomimetic Materials

**Figure 2:**
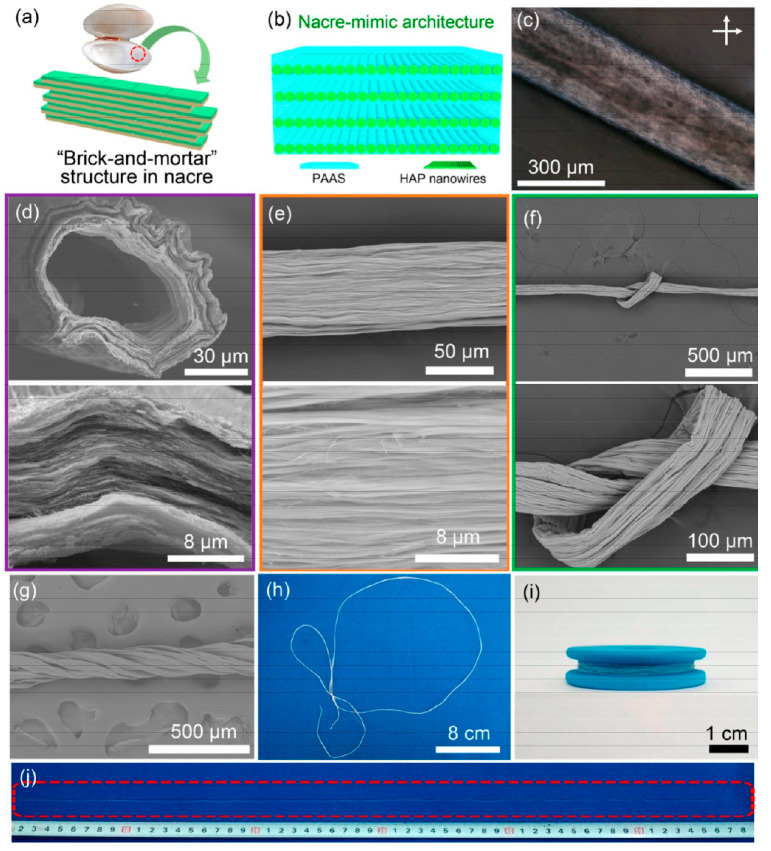
Characterization of the as-prepared HAP/PAAS ribbon fiber. (**a**) “Brick-and-mortar” structure of natural nacre. (**b**) Schematic illustration of the ribbon fiber with a nacre-mimic structure. (**c**) Polarized optical microscope image of the HAP/PAAS gel fiber. (**d**,**e**) SEM images of the cross-section (**d**) and surface morphology (**e**) of the ribbon fiber. (**f**) SEM images of a knot of the ribbon fiber. (**g**) SEM image of a rope prepared by twisting two ribbon fibers. (**h**) Digital image of a rope obtained by twisting 12 ribbon fibers. (**i**) Digital image of a long ribbon fiber collected on a winder. (**j**) Digital image of a long straight ribbon fiber. Reproduced with permission from ref. [40]. Copyright 2018, American Chemical Society.

Nacre is one of the toughest materials in nature, owing to its unique “brick-and-mortar” ordered architecture consisting of high mineral content of aragonite (~95 vol.%) platelets bonded by a thin layer of organic materials. In the “brick-and-mortar” structure, aragonite platelets serve as the “brick” and the biopolymer serves as the “mortar” (Figure 2a). The “brick-and-mortar” structure can withstand high degree of inelastic deformation and efficiently dissipate energy, leading to high toughness up to 10 MPa m^1/2^ [71,72]. Therefore, the “brick-and-mortar” structure of nacre provides a useful bionic model for designing high-performance biomimetic materials.

Inspired by this guideline, efforts have been dedicated to enhance the toughness of biomimetic materials by constructing the “brick-and-mortar” structure [73]. From a mechanical perspective, high toughness is one of essential requirements for materials to possess high strength, because brittle materials often fracture prematurely before reaching theoretical strength and fracture strain values.

Although ultralong HAP nanowires show excellent flexibility at the nano-scale, how to transfer this nano-scale flexibility to the macroscopic scale is a key issue for constructing strong and tough biomimetic materials. To this end, some encouraging studies have been performed. For example, Yang et al. [40] prepared the continuous flexible macroscopic ribbon fibers with a nacre-mimetic “brick-and-mortar” layered structure using highly ordered alignment of ultralong HAP nanowires as the hard “brick” and sodium polyacrylate (PAAS) as the soft “mortar” by a scalable and convenient wet-spinning method (Figure 2b). The quasi-long-range orderly liquid crystal of ultralong HAP nanowires was used and injected by a syringe into absolute ethanol to form the continuous flexible macroscopic ribbon fiber with the nacre-mimetic layered architecture. The HAP/PAAS mixture slurry exhibited a pearl-like brilliant texture and the birefringent optical phenomenon in the polarized optical microscope image, implying the formation of the liquid crystal phase (Figure 2c). The HAP/PAAS ribbon fiber showed the distinct layered structure, mimicking the “brick-and-mortar” structure in natural nacre (Figure 2d). Ultralong HAP nanowires were well aligned along the longitudinal direction of the HAP/PAAS ribbon fiber (Figure 2e). In contrast to the high brittleness of the traditional macroscopic HAP materials, the as-prepared HAP/PAAS ribbon fiber exhibited high flexibility and could be knotted without obvious damage (Figure 2f). A rope could be easily prepared by twisting two HAP/PAAS ribbon fibers together (Figure 2g). In addition, a strong long rope was prepared by twisting 12 HAP/PAAS ribbon fibers together (Figure 2h). A continuous long HAP/PAAS ribbon fiber could be prepared provided that there was a sufficient supply of the HAP/PAAS mixture slurry; a highly flexible and long HAP/PAAS ribbon fiber with a length of tens of meters was made and collected on a winder (Figure 2i), and a highly flexible and straight HAP/PAAS ribbon fiber with a length of nearly 60 cm was prepared (Figure 2j). The as-prepared flexible macroscopic ribbon fiber showed excellent mechanical properties with a maximum tensile strength of 203.58 ± 45.38 MPa and Young’s modulus of 24.56 ± 5.35 GPa. Furthermore, the macroscopic ribbon fiber could be woven into various flexible macroscopic products, and could be functionalized by the incorporation of various functional components, such as magnetic and photoluminescent constituents.

### 4.3. Enamel-and-Nacre-Mimetic Structure: A Novel “Fiberboard-and-Mortar” Structure Facilitates Strong and Tough Biomimetic Materials

As mentioned above, enamel and nacre are almost the strongest and toughest materials, respectively, in nature. It is expected to integrate the structural superiorities of enamel (hierarchical multi-scale ordered nanowire structure) and nacre (“brick-and-mortar” structure) to achieve the novel bioinspired hierarchical multi-scale ordered structure with a combination of high strength and high toughness.

Considering that enamel and nacre are typical examples of natural biomaterials with high strength and high toughness, respectively, the authors’ research group [74] mimicked the structures of both enamel and nacre to construct a new kind of fiberboard-and-mortar hierarchical structure by the multi-scale and multilevel assemblies of ultralong HAP nanowires from the nano-scale to the micro-scale to the macro-scale and from 1-D to 2-D to 3-D. The preparation processes are as follows: (1) ultralong HAP nanowires with diameters of ~10 nm and lengths of several hundred micrometers as the building blocks were prepared by the calcium oleate precursor solvothermal method, and they self-assembled into nanowire bundles along the longitudinal direction (1-D, first level ordering) at the nano-scale; (2) during the injection process of the solvothermal slurry containing ultralong HAP nanowires into ethanol, ultralong HAP nanowire bundles were preferentially oriented along their longitudinal direction to form the macro-scale fiber (1-D, second level ordering), which extended the 1-D alignment structure from the nano-scale to the micro-scale and then to the macro-scale; (3) after the compression molding of parallel aligned macro-scale fibers, macro-scale fibers were assembled into fiberboards, and fiberboards were stacked layer by layer to form the 3-D HAP nanowire bulk sample, realizing the structural transition from 1-D ordering (macro-scale fiber) to 2-D ordering (fiberboard, third level ordering) to 3-D ordering (bulk sample, fourth level ordering); and (4) the polymers (polymethyl methacrylate (PMMA) and polyacrylic acid (PAA)) fill the gaps in the whole framework of the HAP nanowire bulk sample to form the highly ordered ultralong HAP nanowire fiberboard-and-mortar hierarchical structure. The as-prepared fiberboard-and-mortar hierarchical structure contained 75 wt.% ultralong HAP nanowires, which was comparable to that of the natural bone. The as-prepared fiberboard-and-mortar hierarchical structure could avoid the excessively strong interface, which would generate the local stress concentration and brittle failure under high loading, and avoid the too-weak interface, which would significantly reduce the mechanical strength, thus exhibiting enhanced mechanical properties, for example, high strength (308 MPa), high Young’s modulus (34.7 GPa), high toughness (4.77 MPa·m^1/2^), and high hardness (1.32 GPa) [74].

It should be noted that various “mortar” components are applicable to prepare various kinds of fiberboard-and-mortar hierarchical structured biomimetic materials. For example, by replacing the stiff PMMA/PAA with soft PAA, a bioinspired flexible and strong biomimetic hydrogel was prepared [45]. The as-prepared biomimetic hydrogel showed much higher tensile strength (9.33 MPa) than those of conventional hydrogels (usually lower than 1 MPa), and could withstand a weight of 7 kg without fracture.

### 4.4. Compact Bone-Inspired Structure: Mechanically Strong Biomimetic Materials

Bone is the largest HAP-based tissue in the human body, possessing excellent mechanical properties for supporting daily activities. Bone is classified into compact bone (outer layer) and cancellous bone (inner part). In fact, the structure of bone is too complex to be artificially duplicated [75], but studies have revealed that the mechanical properties of materials can be significantly enhanced by mimicking the hierarchical ordered structure of bone [76].

**Figure 3:**
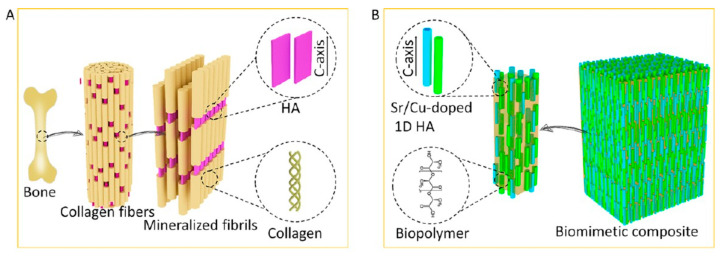
(**A**) The hierarchical ordered structure of natural bone. (**B**) The bone-inspired composite consisting of highly ordered ultralong HAP nanowires. Reproduced with permission from ref. [77]. Copyright 2021, The Authors. Published by American Chemical Society.

Inspired by this guideline derived from bone, Zhang et al. [77] designed a biphasic nanocomposite consisting of highly aligned strontium/copper-doped HAP nanowires and poly(D,L-lactide) (PDLA) (Figure 3). In the biphasic nanocomposite, PDLA formed the organic matrix, and uniformly dispersed, highly aligned Sr/Cu-doped ultralong HAP nanowires acted as an inorganic reinforcement phase and embedded in the PDLA matrix. The biomimetic nanocomposite could release Sr^2+^ and Cu^2+^ ions and support the proliferation and alkaline phosphatase activity of human mesenchymal stromal cells. The oriented Sr/Cu-doped HAP nanowires could induce the alignment of cells throughout the scaffold and formation of anisotropic type I collagen fiber matrix. The biomimetic nanocomposite exhibited a high compressive strength (116 MPa) and high Young’s modulus (6.1 GPa).

### 4.5. Cancellous Bone-Inspired Structure: Highly Elastic and Porous Aerogels

Cancellous bone is the inner part of bone tissue. Different to compact bone, cancellous bone has a honeycomb-like porous structure assembled from interconnected building blocks, and it has high porosities (50–90%) to decrease the weight of bone and provide sufficient room for nerves and blood vessels [78]. Therefore, it is attractive to design lightweight and porous materials inspired by the structure of cancellous bone.

**Figure 4:**
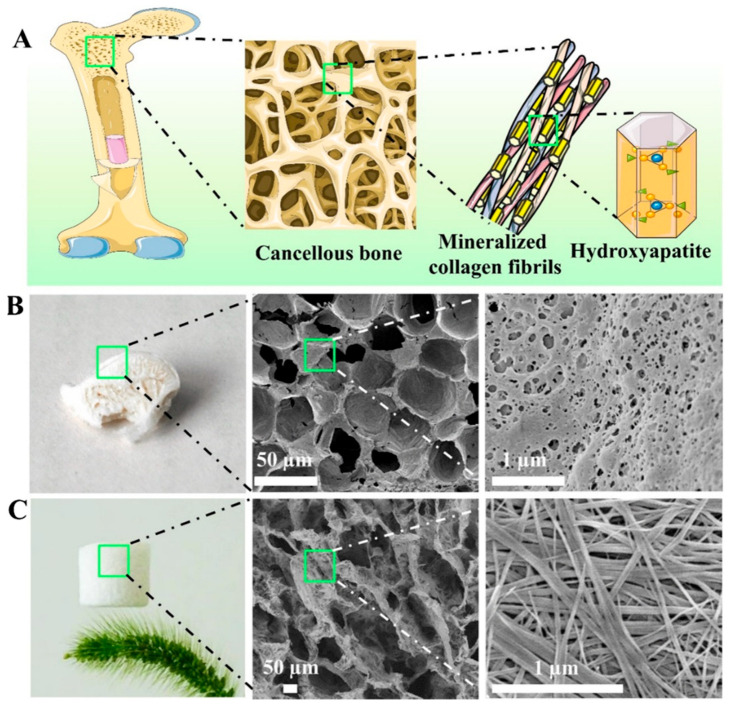
(**A**) Schematic illustration of the structure of cancellous bone. (**B**,**C**) The microstructure of cancellous bone (**B**) and the cancellous bone-inspired aerogel assembled by ultralong HAP nanowires (**C**). Reproduced with permission from ref. [42]. Copyright 2018, American Chemical Society.

Owing to the ultrahigh aspect ratios and ultralong lengths of ultralong HAP nanowires, they can easily interweave into the stable porous network. Inspired by cancellous bone, the authors’ research group prepared a novel kind of aerogel with a cancellous bone-like meshwork architecture using ultralong HAP nanowires as the building blocks (Figure 4) [42]. The HAP nanowire aerogel was prepared by freeze-drying using the hydrothermal product aqueous slurry containing ultralong HAP nanowires. The preparation method is environmentally friendly, low-cost, and can be scaled up for large-scale production. The as-prepared HAP nanowire aerogel showed a highly porous structure, which is similar to the porous meshwork of the cancellous bone at both the nano-scale and the macro-scale. The as-prepared inorganic aerogel exhibited high porosity (~99.7%), ultralight (density 8.54 mg cm^–3^), and superior adiabatic performance (thermal conductivity = 0.0387 W m^–1^ K^–1^). Compared with organic aerogels, the as-prepared HAP nanowire aerogel is biocompatible, environmentally friendly, and low cost. Importantly, the HAP nanowire aerogel showed elasticity, and it could rapidly recover to its original shape and size after removing the load. During the compressing–releasing process of the hydrophobic HAP nanowire aerogel, the maximum compressive strength of the HAP nanowire aerogel increased with the increasing maximum strain. When the load was released, the stress decreased dramatically at the early stage, then decreased slowly, and finally reached zero. The HAP nanowire aerogel exhibited a foam-like elasticity and shape recovery behavior. This work provides a promising structural guideline for the design of highly elastic inorganic aerogel materials.

### 4.6. Mimicking Multi-Layered Concentric Circular Structure of Bone: Favorable Osteogenic and Immune Microenvironment for Bone Regeneration

The ordered arrangement and multi-layered concentric circular structure of natural bone provide unique cues to design high-performance bone-mimetic materials for accelerated bone regeneration. Recently, Geng et al. [79] reported a biomimetically hierarchical hydrogel scaffold with highly ordered arrangement and multi-layered concentric cylinder structure prepared by a convenient free-injection method for osteoimmunomodulation and bone defect repair. In this hierarchical hydrogel scaffold, ultralong HAP nanowires were adopted as the building blocks to assemble into the hierarchically ordered nanowire bundles and further hybridize with hyaluronic acid methacrylate and MgAl-layered double hydroxide nanosheets to form the stable biomimetic scaffold. During the preparation process, a composite bioink containing ultralong HAP nanowire slurry and hyaluronic acid methacrylate was injected into ethanol by a syringe needle, and the strong shear force could induce the parallel alignment of ultralong HAP nanowires to form highly ordered nanowire bundles, which could assemble to form the multi-layered concentric cylinder structure on the transverse section of macroscopic fibers. Finally, the orderly biomimetic macroscopic fibers were parallelly accumulated and cross-linked to form a 3D porous orderly biomimetic scaffold. Moreover, the orderly biomimetic hydrogel scaffold was functionalized with MgAl-layered double hydroxide nanosheets. The biomimetic hydrogel scaffold showed anisotropic structure, high water content, high porosity, good mechanical performance, and sustained release of bioactive Mg^2+^ and Ca^2+^ ions. Importantly, the high biocompatibility, ordered topography, and biochemical cue could guide recruitment and directional migration of bone mesenchymal stem cells and effectively promote osteogenic differentiation and vascularization. In addition, the biomimetic scaffold could induce M2 phenotype polarization of macrophages, creating a favorable osteoimmune environment. The biomimetic scaffold showed reduced inflammation, promoted vascularized ossification, and increased new bone formation in the reported experiments.

### 4.7. Fish Gill-Inspired Multi-Scale Ordered Structure: Enhancing Water–Salt Balance

**Figure 5:**
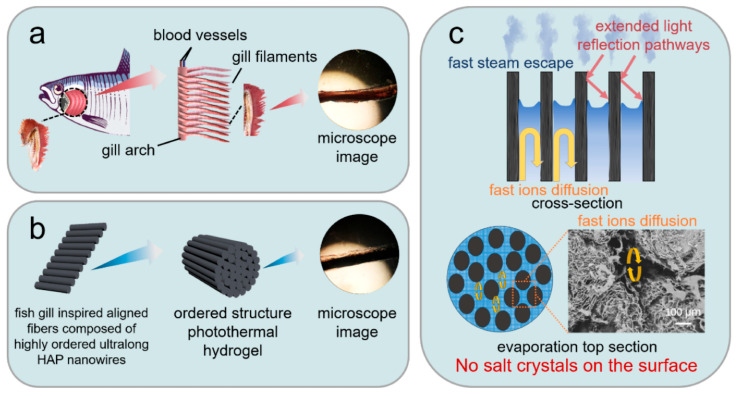
Design of fish gill-inspired biomimetic multi-scale-ordered hydrogel-based solar water evaporator. (**a**) Schematic diagram of the structure of fish gill filaments. (**b**) Schematic diagram of the fish gill-inspired biomimetic multi-scale-ordered hydrogel-based solar water evaporator. (**c**) Schematic illustration of the cross-section and the top evaporating surface of the biomimetic hydrogel-based photothermal solar water evaporator and SEM image of the top evaporating surface. Reproduced with permission from ref. [80]. Copyright 2025, American Chemical Society.

The fish gill is an important tissue for marine fish to maintain the water–salt balance. The gill lamellae of fish are composed of many ordered gill filaments that separate the internal environment from the surrounding water, providing a large surface for the diffusion of gases and salts, and the gills are the primary site for salt flow control in the fish (Figure 5a). Fish can regulate osmotic pressure by returning salt ions through pore channels between orderly gill filaments and by physiological changes.

Inspired by this guideline, Chen et al. [80] developed a biomimetic hydrogel composed of highly ordered ultralong HAP nanowires, MXene, and hydrophilic hyaluronic acid methacryloyl, and it had a multi-scale-ordered hierarchical architecture which mimicked the fish gill structure. The highly ordered alignment of ultralong HAP nanowires was realized at multiple scales, from the nano-scale to the micro-scale to the macro-scale and from 1D to 2D to 3D in the biomimetic hydrogel. The biomimetic hydrogel could be used as a solar water evaporator for efficient seawater desalination (Figure 5). The as-prepared biomimetic hydrogel water evaporator showed high efficiency of photothermal conversion, low water evaporation enthalpy, excellent heat management capability, and high solar water evaporation performance. The water evaporation enthalpy reduced from 2431 J g^−1^ (pure water) to 1113 J g^−1^. As a result, the biomimetic hydrogel water evaporator exhibited a high water evaporation rate (6.278 kg m^−2^ h^−1^) and a high energy conversion efficiency (129% for pure water) under one sun illumination (1 kW m^−2^). Furthermore, owing to the ordered channels between ordered ultralong HAP nanowires that could accelerate the salt–water exchange and promote the salt-ion reflux, the as-prepared biomimetic hydrogel evaporator could achieve a water evaporation rate of 4.931 kg m^−2^ h^−1^ using real seawater samples. The water evaporation rate was maintained at 3.109 kg m^−2^ h^−1^ even in a saturated NaCl solution for at least 10 h without significant degradation, and no solid salt deposit was observed on the evaporation surface.

### 4.8. Plant-Inspired Vertically Aligned Channel Structure: Enhanced Water Evaporation

**Figure 6:**
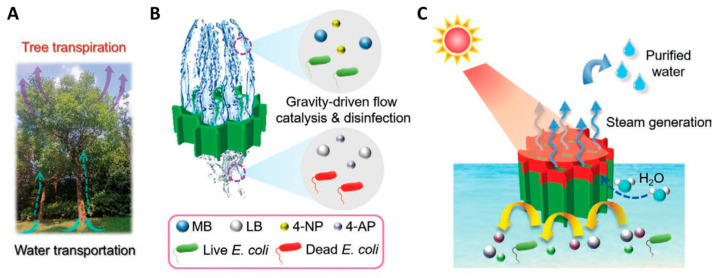
Schematic illustration of the tree-inspired biomimetic aerogel with vertically aligned channels. (**A**) Water transportation and transpiration of natural tree. (**B**) Multiple functions of the biomimetic aerogel in gravity-driven continuous flow catalysis and water disinfection. (**C**) Solar energy-driven water evaporation for seawater desalination and wastewater purification. Reproduced with permission from ref. [84]. Copyright 2021, Wiley-VCH.

Natural plants with unique structures and properties provide inspirations to create advanced structural and functional materials. For example, tree transpiration is a natural process, wherein water transports from the bottom root up to leaves through xylem vessels with vertically aligned channels inside tree trunks and branches (Figure 6A), and the water vapor releases to the surroundings [81,82,83].

Inspired by water transportation and transpiration of natural trees, the authors’ research group [84] developed a biomimetic aerogel with vertically aligned channels and multiple functions for solar energy-driven seawater desalination, water purification, water disinfection, and continuous flow catalysis (Figure 6). In this biomimetic aerogel, ultralong HAP nanowires were used as flexible building blocks for the skeleton framework and hybridized with chitosan and palladium catalyst nanoparticles. The biomimetic aerogel was prepared by a unidirectional freeze-drying method using Pd nanoparticles decorated ultralong HAP nanowires/chitosan. Owing to the interconnected cellular structure, unidirectional aligned channels, nanowire-interwoven networked pore wall, and evenly distributed Pd nanoparticles, the biomimetic aerogel showed a high catalytic activity (97.6%) and permeability (1786 L m^−2^ h^−1^), excellent recyclability, high stability in the continuous flow catalytic degradation of methylene blue, high performance in bacteria removal and anti-biofouling, and high efficiency for solar energy-driven seawater desalination and wastewater purification.

Lotuses can survive in heavy storms although long and slender lotus stems possess hierarchical porous architecture with 12 or 13 well cross-section structure along lotus stems [85]. The lotus stem has a parallel arranged vascular tissue composed of macroporous vascular bundles in the longitudinal direction, which can transport water and mineral nutrients upward from the root.

Inspired by the long-range ordered structure and water transportation capability of lotus stem, Wang et al. [86] prepared a biomimetic aerogel with vertically ordered channels and low water evaporation enthalpy for high-performance solar energy-driven salt-resistant seawater desalination and wastewater purification. The as-prepared biomimetic aerogel was composed of ultralong HAP nanowires as the heat-insulating skeleton, polydopamine-modified MXene as a photothermal material, and polyacrylamide and polyvinyl alcohol as reagents to lower the water evaporation enthalpy and as glues to enhance the mechanical performance. Owing to the honeycomb porous structure, unidirectionally aligned channels, and nanowire/nanosheet/polymer pore walls, the biomimetic aerogel exhibited superior mechanical properties, high water absorption capacity (22.4 g g^−1^), rapid water transportation, high sunlight absorption efficiency (96.2%), high solar water evaporation rate (2.62 kg m^−2^ h^−1^) under one sun illumination, and low water evaporation enthalpy (1425.61 J g^−1^). Furthermore, the biomimetic aerogel showed parallel channels with low tortuosity, where water could transport upward from the bottom and the salt ions on the top surface of the aerogel could diffuse through parallel channels downward into bulk water, leading to the inhibition of salt deposition on the water evaporating surface.

## 5. Assembly Strategies of Flexible Ultralong HAP Nanowires for Biomimetic Ordered Structures

Inspired by the design principles and structure-and-property relationships of natural materials, many biomimetic materials with hierarchical ordered structures based on flexible ultralong HAP nanowires have been developed and they exhibit superior properties and promising applications in various fields. In this section, we discuss the strategies for designing and assembling flexible ultralong HAP nanowires into biomimetic hierarchical ordered structures. These assembly strategies are also applicable to the construction of other high-performance bioinspired materials.

### 5.1. Ultralong HAP Nanowire Liquid Crystal

At the nano-scale, ultralong HAP nanowires tend to self-assemble into nanowire bundles along the longitudinal direction. In order to transfer this ordering manner from the nano-scale to the macro-scale, one efficient strategy is to prepare a kind of quasi-long-range orderly liquid crystal containing ultralong HAP nanowires.

We have found that ultralong HAP nanowires exhibit liquid crystal behavior in the product slurry obtained by the calcium oleate precursor solvothermal method [40]. The as-prepared ultralong HAP nanowires are highly anisotropic and are able to form a quasi-long-range orderly liquid crystal phase. In the solvothermal product slurry, ultralong HAP nanowires with very high aspect ratios (>10,000) can align along the longitudinal direction, leading to the formation of the locally ordered structure [39]. The oleate groups can enhance interactions between ultralong HAP nanowires, promoting the formation of locally ordered structures and enabling their stable dispersion. This phenomenon can be explained by Onsager’s theory [87]. The 1-D structures with high aspect ratios tend to arrange in parallel spontaneously to form the locally ordered structure above a critical concentration because of the excluded volume effect. The excluded volumes of 1-D structures with high aspect ratios are so large that they overlap at high concentrations, which restricts the degree of freedom for 1-D structures, leading to entropy loss. The system is thermodynamically stable when 1-D structures arrange in parallel. The increase in the positional entropy compensates the loss of orientational entropy caused by the ordering of 1-D structures, and enhanced total entropy produces a quasi-long-range orderly liquid crystal phase [40].

**Figure 7:**
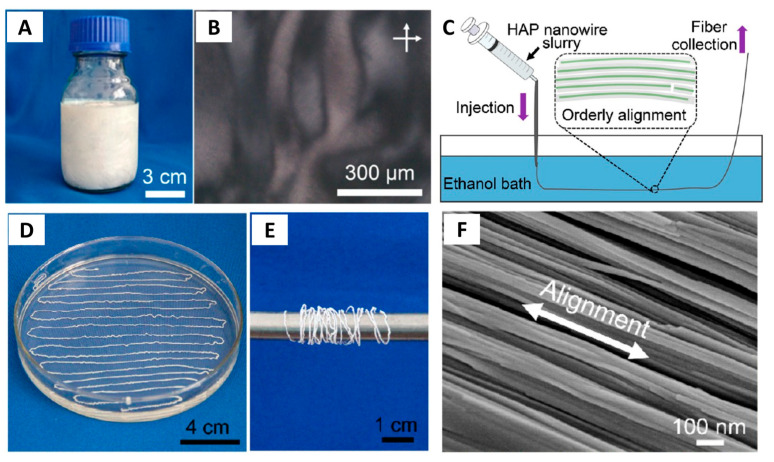
(**A**) Digital image of the solvothermal product slurry containing ultralong HAP nanowires. (**B**) Polarized optical microscopy image of the solvothermal product slurry containing ultralong HAP nanowires. (**C**) Schematic illustration of the preparation of the macroscopic HAP nanowire ribbon fiber by the injection method. (**D**,**E**) Digital images of the macroscopic ribbon fiber consisting of highly ordered ultralong HAP nanowires. (**F**) SEM image of the macroscopic ribbon fiber, in which ultralong HAP nanowires are highly aligned along the longitudinal direction. Reproduced with permission from ref. [40]. Copyright 2018, American Chemical Society.

The solvothermal product slurry containing ultralong HAP nanowires showed a pearl-like brilliant texture (Figure 7A), implying the formation of the liquid crystal phase. The polarized optical microscope was used to observe the solvothermal product slurry containing ultralong HAP nanowires, and the birefringent optical texture was observed in the image of the solvothermal product slurry (Figure 7B), suggesting the local orientation of ultralong HAP nanowires and the formation of the liquid crystal phase [40].

### 5.2. Wet Spinning (Injection) Method

Large-sized highly ordered HAP nanostructures are of great significance for applications in various fields and for understanding the formation mechanisms of bone and tooth. However, the synthesis of large-sized highly ordered HAP nanostructures remains a great challenge, especially for the preparation of large-sized highly ordered ultralong HAP nanowires because ultralong HAP nanowires are easily tangled and aggregated. Owing to unique properties of the quasi-long-range orderly structure, the liquid crystalline ultralong HAP nanowires can be used to prepare highly ordered architectures and composite materials by hybridization with polymers. In 2016, the authors’ research group [39] discovered for the first time the ability of the solvothermal product slurry containing ultralong HAP nanowires prepared by the calcium oleate precursor solvothermal method to be assembled into macroscopic fibrous materials with the long-range ordered structure via the wet spinning (injection) method. This strategy is highly efficient for the rapid automated production of highly flexible, large-sized, self-assembled highly ordered ultralong HAP nanowires at room temperature and their derived materials, such as high-strength highly flexible nanostructured ropes (nanoropes), highly flexible textiles, and 3-D printed well-defined highly ordered patterns. These highly ordered nanostructures are successively formed from the nano-scale to the micro-scale then to the macro-scale, and the ordering direction of ultralong HAP nanowires is controllable.

In a subsequent work of the authors’ research group, the wet spinning (injection) method was used to prepare the highly ordered macroscopic ribbon fiber that could inherit the orderly structure of the liquid crystalline HAP nanowire slurry (Figure 7C) [40]. The solvothermal product slurry containing ultralong HAP nanowires was loaded into a syringe and slowly injected into ethanol. The continuous and long macroscopic ribbon fiber consisting of highly ordered ultralong HAP nanowires and with a desired length could be obtained as long as the solvothermal product slurry was sufficiently provided. For example, a macroscopic ribbon fiber composed of highly ordered ultralong HAP nanowires with a length of several meters was prepared, and it exhibited a high flexibility and could be wound around a metal rod (Figure 7D–F).

During the wet spinning process, the shear force from the syringe induces the aligned arrangement of ultralong HAP nanowires. This assembly strategy is highly efficient when the liquid crystal phase is used, which usually forms flexible macroscopic fibrous materials with the long-range ordered structure. One of the benefits of wet spinning for ultralong HAP nanowire orderly assembly is that the length and diameter of the product can be easily controlled by the movement distance and diameter of the syringe. Theoretically, the macroscopic fibers with the long-range continuously ordered structure prepared by wet spinning can be infinitely long, enabling them to be further woven into various shapes [39].

**Figure 8:**
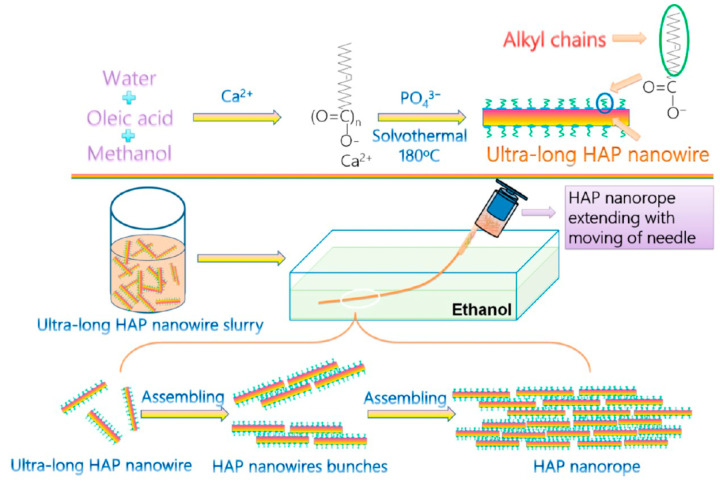
Formation mechanism of orderly assembly of ultralong HAP nanowires with the long-range ordered structure by the wet spinning method. Reproduced with permission from ref. [39]. Copyright 2016, American Chemical Society.

We proposed a surface-induced instant self-assembly process for the mechanism of the orderly assembly of ultralong HAP nanowires during the wet spinning process (Figure 8) [39]. In the reaction system, oleic acid molecules can adsorb onto the surface of ultralong HAP nanowires through interactions between carboxyl groups of oleic acid molecules and Ca^2+^ ions of ultralong HAP nanowires, with their alkyl chains exposed to the mixed solvent. When the solvothermal product slurry containing ultralong HAP nanowires is injected into anhydrous ethanol, the injection shear force induces ultralong HAP nanowires to align directionally parallel to each other. The components of water, methanol (or ethanol), and oleic acid molecules in the solvothermal product slurry diffuse into ethanol. Because alkyl chains of oleic acid molecules are incompatible with the polar solvent (ethanol, methanol, and water), ultralong HAP nanowires are separated out from the solvent. During the process of solvent diffusion, oleic acid molecules adsorbed on the surface of ultralong HAP nanowires may modulate the self-assembly process of ultralong HAP nanowires by the “alcohol–oleic acid” interaction, expelling ultralong HAP nanowires to form aligned nanowire bundles. Because HAP nanowires have ultralong lengths, each ultralong HAP nanowire may exist in different aligned nanowire bundles. Therefore, ultralong HAP nanowires are linked to each other by this connection, instantly forming the macroscopic fibers with the long-range continuously ordered structure [39].

The wet spinning strategy can be combined with homemade automated equipment using round-end needles, which enables the rapid automated production of high flexible macroscopic fibers with the long-range continuously ordered structure based on ultralong HAP nanowires [39]. Moreover, the introduction of polymers such as sodium polyacrylate could significantly enhance the mechanical properties of the macroscopic fibers with the highly ordered structure. The as-prepared flexible macroscopic HAP/sodium polyacrylate ribbon fiber showed superior mechanical properties, and the maximum tensile strength was as high as 203.58 ± 45.38 MPa [40], much higher than that of a pure single HAP macroscopic fiber (40.1 MPa) [39].

### 5.3. Freeze-Drying Method

In recent years, the freeze-drying method has become one of the most popular strategies for material preparation and assembly [88,89]. In particular, it is applicable to the assembly of many inorganic building blocks because they are usually insoluble in water. This strategy includes the dispersion of building blocks in water, followed by a controlled freezing process (via cooling rate, temperature gradient, etc.) to induce ice crystal formation [90,91]. The building blocks fill interlayer voids between ice crystals. After that, the frozen sample undergoes sublimation treatment, during which ice crystals are removed to form materials with the porous structure [92].

The underlying mechanism of the freeze-drying method is to use grow ice crystals to dynamically construct the adjustable microstructures. When water nucleates and freezes, ice crystals form. The growing ice crystals repel non-aqueous phases (such as polymer monomers dissolved in water, colloidal nanoparticles dispersed in water, and insoluble substances mixed in water) to the surrounding area. Controlling the growth of ice crystals by physical and chemical methods can prepare the desired hierarchical ice structures, which can retain the inverse structures of ice crystals after removing the ice crystals [88].

The traditional non-directional freeze-drying method is feasible to fabricate cancellous bone-mimetic interconnected porous materials based on ultralong HAP nanowires. One advantage of the non-directional freeze-drying method is that it does not require controlling the growth direction of ice crystals [93]. This method facilitates the preparation of large-sized, porous materials with various shapes (depending on the shape and size of the mold).

Inspired by the porous structure of cancellous bone, Zhang et al. [42] prepared a biomimetic inorganic aerogel composed of ultralong HAP nanowires by the non-directional freeze-drying method. The as-prepared ultralong HAP nanowire aerogel exhibited excellent properties such as excellent elasticity, high porosity (≈99.7%), very low density (8.54 mg cm^–3^), and low thermal conductivity (0.0387 W m^–1^ K^–1^). Huang et al. [94] prepared a highly porous elastic biomimetic aerogel scaffold made from flexible ultralong HAP nanowires by the non-directional freeze-drying method for application in bone regeneration. In this biomimetic aerogel scaffold, flexible ultralong HAP nanowires were highly interwoven to form the walls of the aerogel.

During the freezing process, ice crystals nucleate and grow on the cold surface, and the nucleation and growth of ice crystals can be controlled along the desired directions [88]. The directional freeze-drying method can be realized by controlling the temperature gradient of freezing and directional nucleation and growth of ice crystals [95,96]. Inspired by the structure of natural trees, Xiong et al. prepared a multifunctional biomimetic aerogel with vertically aligned channels using ultralong HAP nanowires by the unidirectional freeze-drying method [84]. During the preparation process, ultralong HAP nanowires were dispersed in deionized water. Then, ultralong HAP nanowire aqueous suspension, chitosan aqueous solution, acetic acid, and poly(ethylene glycol) diglycidyl ether were mixed under stirring. Subsequently, the homogeneous suspension was poured into a cylindrical mold, followed by a directional freezing process, in which the mold was placed on a cold platform in contract with liquid nitrogen as the cold source. The surrounding surface of the mold was insulated to ensure the unidirectional freezing direction from the cold platform. After that, the frozen sample was dried in vacuum by the freeze drier to obtain the ultralong HAP nanowire aerogel with the vertically aligned channel structure. Finally, the aerogel was thermally cross-linked.

The bi-directional freezing mode can induce the growth of ice crystals in both vertical and horizontal directions. If a wedge with a specific angle and low thermal conductivity is introduced between the liquid phase and the metal substrate, dual temperature gradients in both vertical and horizontal directions can be produced, leading to a laminar porous structure [97]. As an example, ultralong HAP nanowires and the polymer occupy the gaps between ice lamellae during the freezing process. After the sublimation treatment, lamellae ice crystals are removed to form the ordered lamellae structure, ultimately achieving the parallel orientation of ultralong HAP nanowires.

Zhao et al. [69] reported a biomimetic hierarchical enamel analog at multiple scales by the assembly of amorphous intergranular phase (amorphous ZrO_2_)-coated ultralong hydroxyapatite nanowires. The macroscopic nanocomposite with a parallel arrangement of the nanowires was prepared by dual-directional freezing of the nanowire composite dispersion in the presence of polyvinyl alcohol. In their experiment, the polydimethylsiloxane wedge was used to form bi-directional temperature gradients, resulting in ice crystal growth in both vertical and horizontal directions. The vertical growth of ice crystals forced the nanowires and polyvinyl alcohol to occupy the gaps between ice lamellae, and the horizontal ice growth forced them to form a parallel orientation. After freeze-drying and mechanical compression, dense artificial tooth enamel was obtained.

### 5.4. Three-Dimensional Printing Strategy

Three-dimensional printing is an emergent technique to fabricate materials with elaborate microstructures and complex shapes. Three-dimensional printing enables the layer-by-layer manufacturing of materials with complex geometries directed by computer-aided digital models in an efficient and precise manner. Currently, three-dimentional printing has become promising a tool for building various engineered bulk materials with meticulously crafted architectures through point-by-point, line-by-line, or layer-by-layer localized forming mechanisms [98,99]. Three-dimensional printing has attracted considerable attention because it can design and fabricate many novel materials with tailored and complex structures that are difficult to achieve by other conventional methods [100,101,102].

Currently, three categories of three-dimentional printing are commonly used for multi-scale and multimaterial fabrication, including material extrusion, photopolymerization, and energy deposition. Material extrusion is a type of mass delivery process whereby slurry or paste inks are squeezed through small nozzles to form droplets or ligaments that can be hardened through rheological or thermal changes, including ink jetting, direct ink writing, and fused deposition modeling. Extrusion-based three-dimentional printing can be applied to a wide variety of materials by developing printable inks and tuning the printing parameters. Photopolymerization is a class of selective curing processes using a light source on photosensitive resins to induce polymerization at specific positions. Energy deposition involves applying a high-power laser or electron beam to irradiate a specific point or area to locally melt these materials together and form three-dimentional structures [98].

**Figure 9:**
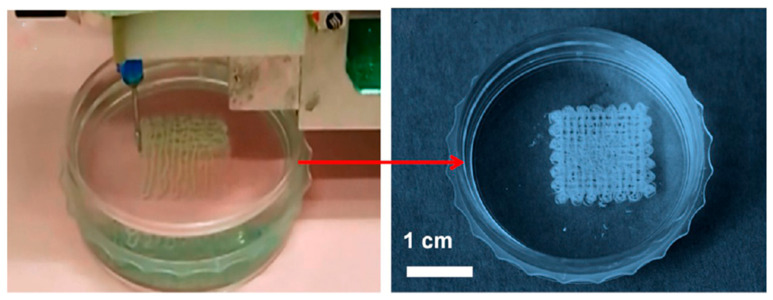
The highly ordered three-dimentional bulk material with the biomimetic multi-scale hierarchical ordered structure and well-defined pattern prepared using ultralong HAP nanowires by three-dimentional printing. Reproduced with permission from ref. [39]. Copyright 2016, American Chemical Society.

With the above merits, efforts have been dedicated to prepare ultralong HAP nanowire-based biomimetic materials with complex shapes and microstructures. More importantly, due to the quasi-long-range orderly liquid crystal behavior of flexible ultralong HAP nanowire slurry prepared by the calcium oleate precursor solvothermal method, the shear force from the three-dimentional printer nozzle can induce the aligned arrangement of ultralong HAP nanowires. This facilitates the formation of biomimetic multi-scale hierarchical ordered structures with well-controlled shapes. In a typical example, the authors’ research group demonstrated the feasibility of ultralong HAP nanowires for the fabrication of the well-defined highly ordered three-dimentional materials with biomimetic multi-scale hierarchically ordered structures and well-defined patterns using a commercial three-dimentional printer (Figure 9) [39].

### 5.5. Multistage Bottom-Up Assembly

It is well-known that natural materials undergo lengthy multistage bottom-up assembly processes to form complex hierarchical ordered structures. Biomineralized tissues show complex hierarchical ordered structures at multiple length scales derived from the bottom-up self-assembly processes; they use a bottom-up approach to specifically tailor their structures and properties, involving the spatial organization of amorphous precursors regulated by biomolecules and the self-assembly of nano-scale biomineral building units into hierarchical ordered structures. The bottom-up assembly method is related to the formation of multifunctional nanostructured materials by the self-assembly of atoms, molecules, or nanostructures. The bottom-up method is useful in the construction of complex nanostructures that are difficult to achieve by the top-down strategy. However, in the preparation of many artificial materials by the bottom-up strategy, only the structures at the nano-scale along one direction can be controlled. Another problem is the lengthy assembly process. Biomineralized tissues usually require a long period of time (several to dozens of years) to construct and grow their structures, which is unworthy and unpractical for the production of artificial materials [3].

Based on this inspiration, the biomimetic hierarchical ordered materials can be prepared by rationally designing and controlling the orderly assembly behaviors of building blocks step-by-step. In general, the sole self-assembly of building blocks can only achieve ordered structures at the nano-scale or the micro-scale, but it is difficult to further assemble them into macro-scale ordered structural materials. For instance, ultralong HAP nanowires can only self-assemble into nanowire bundles along the longitudinal direction with diameters of hundreds of nanometers in the HAP nanowire liquid crystal slurry. Relying on a single assembly strategy, such as wet spinning, can only assemble these microscopically self-organized liquid crystal nanowires into macroscopically 1-D ordered fibers and cannot achieve 3-D multi-level hierarchical ordered structures. Fortunately, 1-D ordered macroscopic fibers are able to assemble into biomimetic 3-D materials with the assistance of additional assembly strategies. Consequently, 3-D ultralong HAP nanowire-based biomimetic materials with hierarchical ordered architectures can be constructed by multiple assembly methods.

For example, inspired by dental enamel, a multi-scale bottom-up assembly strategy was developed to construct the dental enamel-mimic structural materials using highly ordered ultralong HAP nanowires reinforced with resin [64]. By aligning the 1-D HAP macroscopic fibers composed of highly aligned ultralong HAP nanowires into a well-designed mold, followed by a polymerization process, 3-D biomimetic materials with a dental enamel-mimetic structure can be obtained, achieving structural transition from 1-D ordering to 3-D hierarchical and multi-scale ordering. In the multi-scale bottom-up assembly processes, the multi-scale (from the nano-scale to the micro-scale to the macro-scale) highly ordered alignment structure similar to that in the dental enamel could be achieved.

On this basis, if the parallel aligned HAP macroscopic fibers are compressively molded into the 3-D HAP nanowire bulk consisting of orderly layered nanowire fiberboards, the structural transition can be realized from 1-D ordering (macroscopic fiber) to 2-D ordering (ultralong HAP nanowire fiberboard) to 3-D hierarchical ordering (HAP nanowire bulk) [74]. This method combined the structural merits of both enamel (highly ordered bundles) and nacre (brick-and-mortar structure) to construct the fiberboard-and-mortar alignment hierarchical structure consisting of highly ordered ultralong HAP nanowires by the multi-scale and multilevel assembly strategies. The as-prepared biomimetic materials with the fiberboard-and-mortar alignment hierarchical structure exhibited superior mechanical properties such as high strength, high Young’s modulus, and high toughness [74].

## 6. Applications of Ultralong HAP Nanowire-Based Biomimetic Materials

With the “strong-and-flexible” characteristic of ultralong HAP nanowires and derived biomimetic materials with the hierarchical ordered structures, ultralong HAP nanowire-based biomimetic materials show promising applications in various fields, such as mechanical protection, heating insulation, flexible electronic devices, medical treatment, and solar energy-driven seawater desalination.

### 6.1. Mechanical Applications

Strong and tough structural materials are highly desirable in mechanical applications. Different from the functional materials, the studies on structural materials mainly focus on mechanical properties because these materials are usually subjected to loads and forces in service. Inorganic nonmetal oxide materials are good candidates for the construction of structural materials owing to their high strength, high modulus, high temperature resistance, and high stability. One challenge for inorganic nonmetal oxide structural materials is their high brittleness, which usually results in poor reliability for the application in the mechanical support and protection.

In recent years, the development of flexible ultralong HAP nanowires as well as bioinspired hierarchical ordered structures may solve the problem of high brittleness. It is important to design and prepare strong and tough bioinspired structural materials with hierarchical ordered structures based on ultralong HAP nanowires.

The ideal engineering structural materials should possess high strength, high toughness, high modulus, lightweight, and high damping effect for energy absorption. HAP is the primary inorganic component of hard tissues such as bone and teeth, which exhibit exceptional mechanical properties to provide protection and support functions. Ultralong HAP nanowires possess high strength, high modulus, and good flexibility. By mimicking the structures of enamel and nacre, the fiberboard-and-mortar structural materials based on highly ordered flexible ultralong HAP nanowires were prepared, which exhibited excellent mechanical properties such as lightweight, high strength (308 MPa), high Young’s modulus (34.7 GPa), high toughness (4.77 MPa m^1/2^), and good durability, which are much better than other HAP-based materials and many engineering materials reported in the literature. The fiberboard-and-mortar structural materials can well trade off strength and toughness of engineering materials—namely, high-strength materials are often brittle, while tough materials tend to deform easily and thus exhibit low strength [3]. Thus, fiberboard-and-mortar materials are promising for application as structural materials and engineering materials such as safety protection.

As demonstrated in an experiment, a 200 g weight was dropped in free fall from a 60 mm height above the sample. Alumina ceramic and polymethyl methacrylate were adopted as control samples. The alumina ceramic with a high strength was instantly broken after being hit. The polymethyl methacrylate sample, a commercial organic glass with high strength and toughness, broke into pieces by hitting. In contrast, the fiberboard-and-mortar structural material based on highly ordered ultralong HAP nanowires was intact without damage after the dynamic impact. The high impact-tolerant ability of the fiberboard-and-mortar structural materials based on highly ordered ultralong HAP nanowires is promising for various applications such as safety protection for human lives and valuables [74].

The viscoelastic properties of materials are characterized by the viscoelastic figure of merit (VFOM), which is the product of storage or Young’s modulus (GPa) and damping coefficient. The VFOM values of traditional solid materials are usually lower than 0.6. However, the VFOM value of the fiberboard-and-mortar structural materials based on highly ordered ultralong HAP nanowires was as high as 0.9 at room temperature, indicating its superior durability for long-term shocks and environmental vibrations, avoiding premature and catastrophic failure, which is desirable for practical applications [74].

Dynamic mechanical analysis indicated that the storage modulus (E′) was high, which describes the load-bearing capacity, stiffness, and deformation resistance of the materials. The E′ values were high at temperatures below 80 °C, and then decreased significantly at higher temperatures because of the glass transition of polymers, indicating that the fiberboard-and-mortar structural materials are strong and stiff enough to endure the fierce shock in a relatively wide temperature range. In addition, the loss modulus (E″) values, which are associated with the energy dissipated capacity caused by the viscous deformation of materials, were also high in a relatively broad temperature range, which can be explained by the multiple energy absorption and dissipation in the “fiberboard-and-mortar” structure. The damping behaviors of materials are commonly characterized by the loss factor (tan δ), which is equal to the loss modulus divided by the storage modulus. The tan δ values of the fiberboard-and-mortar structural materials were in a range from 0.02 to 0.13 in the temperature range for testing, indicating that the materials exhibit relatively good damping properties in a wide range of working temperature, which can be used for shock reduction, sound absorption, etc. Many materials have only high loss factor or high storage modulus values, and have difficulty supporting high loads or transmit vibrations. In contrast, the fiberboard-and-mortar structural materials exhibit high values of both storage modulus and loss factor with good impact-tolerant and impact-decaying abilities [74].

In another work, the authors’ research group [45] developed a flexible, high-strength, and versatile hydrogel with a fiberboard-and-mortar hierarchically ordered structure based on highly ordered ultralong HAP nanowires and polyacrylic acid with a high water content (~70 wt.%), dense structure, and excellent mechanical properties. The as-prepared fiberboard-and-mortar ordered structured hydrogel had abundant ordered water channels, and could be used for the loading, release, and directed delivery of various functional substances. In addition, the hierarchically ordered structure of the hydrogel allowed for multiple energy dissipation pathways, such as the nanowire fracture, nanowire pull-out, deformation, and crack bridge after fracture, which could significantly decrease the stress concentration. The strength and Young’s modulus were enhanced to 9.33 MPa and 277 MPa, respectively. In comparison, the strength values of most traditional hydrogels are usually smaller than 1 MPa.

### 6.2. Thermal Insulation

Many inorganic nonmetal oxide materials show fire resistance, low thermal conductivity, and good heat insulation performance. In order to enhance heat insulation performance, appropriate structures are essential. Owing to the relatively large intermolecular distances, the thermal conductivities of gases are significantly lower than those of liquids and solids [103]. Therefore, the thermal conductivity of materials can be decreased by the introduction of the porous structure. In other words, the high porosity of materials favors the thermal insulation performance. Based on this strategy, many porous materials have been developed for thermal insulation application, focusing on enhancing the porosity of materials to reduce thermal conductivity. However, excessively high porosity compromises structural integrity, thereby diminishing the material’s mechanical properties and undermining the stable performance. Consequently, superior heat insulation materials require rational designs of porous structures to address the trade-off between mechanical properties and thermal conductivity. In addition, the thermal stability represents another critical consideration for the heat insulation ability of materials. In porous materials, organic compounds with poor thermal stability are often required as the glue for structural reinforcement. However, this also compromises the thermal stability of composite materials, reducing their working temperature range.

Ultralong HAP nanowires and their derived materials show high thermal stability, nonflammability, and low heat conductivity [26,27]. In order to enhance the thermal insulation performance, the authors’ research group designed and prepared a cancellous bone-inspired aerogel consisting of exclusive ultralong HAP nanowires without adding any polymer [42]. More importantly, ultralong HAP nanowires can form a highly stable porous structure without the addition of the organic glue because of their ultrahigh aspect ratios and ultralong sizes. Owing to the high melting point (~1650 °C) of HAP, the ultralong HAP nanowire aerogel exhibited high thermal stability when it was exposed to high temperatures for a long time. The ultralong HAP nanowire aerogel possessed an ultrahigh porosity of 99.7%, and thus it was highly adiabatic, with a thermal conductivity as low as 0.0387 W m^–1^ K^–1^.

**Figure 10:**
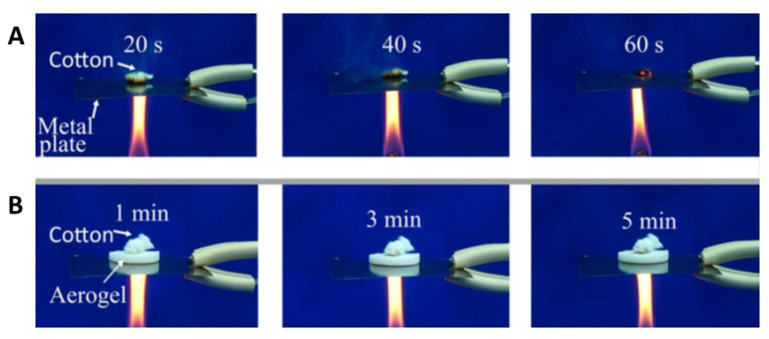
(**A**) A piece of cotton is placed on a metal plate heated by an alcohol lamp, and the cotton is burnt within 1 min. (**B**) A piece of cotton can be well-protected by the ultralong HAP nanowire aerogel (thickness = 5 mm) from the flame of an alcohol lamp even after heating for 5 min. Reproduced with permission from ref. [42]. Copyright 2018, American Chemical Society.

Because the highly porous materials can efficiently restrain heat diffusion, the ultralong HAP nanowire aerogel showed superior thermal insulation performance. As shown in Figure 10, when a piece of cotton was directly placed on a metal sheet in contact with the flame of an alcohol lamp, the cotton was burnt and carbonized rapidly within 1 min. However, a piece of cotton can be well-protected by the ultralong HAP nanowire aerogel as a heat insulator (thickness = 5 mm) from the flame of an alcohol lamp even after heating for 5 min. The infrared image of the ultralong HAP nanowire aerogel (thickness = 1 cm) on a metal plate after heating by an alcohol lamp for 60 min indicated that the temperature gradually decreased from 430 °C at the bottom to 55 °C on the top of the aerogel, revealing its superior thermal insulation performance [42].

### 6.3. Flexible and Knittable Materials

One of the advantages of fiber materials is their knittability. For example, many fiber materials such as metal nanowires, polymer fibers, and carbon fibers have been woven into smart fiber devices with tailored functionalities [104]. However, the knittability of inorganic nonmetal oxide materials is poor because of their high brittleness and easy fracture.

**Figure 11:**
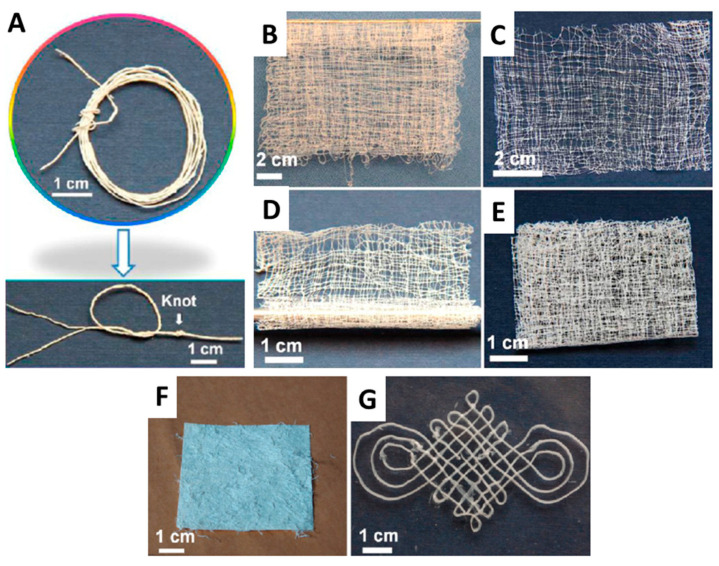
Digital images. (**A**) A highly flexible fire-resistant nanorope consisting of highly ordered ultralong HAP nanowires. (**B**–**E**) The highly flexible fire-resistant gauze-like textiles made from the nanoropes consisting of highly ordered ultralong HAP nanowires in a spreading, rolled, or folded state. (**F**) A nonwoven textile made from the nanoropes consisting of highly ordered ultralong HAP nanowires. (**G**) A complex well-designed pattern made from the nanoropes consisting of highly ordered ultralong HAP nanowires. Reproduced with permission from ref. [39]. Copyright 2016, American Chemical Society.

Owing to the high flexibility of ultralong HAP nanowires, the nanoropes (macroscopic fibers) (Figure 11A) consisting of highly aligned flexible ultralong HAP nanowires show a high flexibility, high strength, and high toughness, which can be woven into functional flexible fabrics with superior mechanical properties. The tensile strength of a single nanorope consisting of highly ordered ultralong HAP nanowires was measured to be 40.1 MPa, and its tensile strength could be further enhanced by twisting a number of nanoropes together. The tensile strength obviously increased when the number of twisted single nanoropes increased from 1 to 3, which were 40.1 MPa, 65.8 MPa, and 105.8 MPa, respectively. Furthermore, a combined nanorope by twisting 36 single nanoropes could withstand a weight of 500 g load without breaking. The high flexibility of the combined nanorope could be preserved, and it could be bent and tied into a knot [39].

The highly flexible fire-resistant gauze-like textiles made from the nanoropes consisting of highly aligned ultralong HAP nanowires were prepared by crisscross moving the injecting needles at room temperature (Figure 11B–E). By crisscross moving the injection directions of the ultralong HAP nanowire slurry into ethanol, a flexible fire-resistant textile composed of highly aligned ultralong HAP nanowires was prepared, and the patterns of the textile could be controlled by the moving mode of the injection needle. The textiles could be rolled and folded without fracture. In addition, a nonwoven textile made from the nanoropes consisting of highly aligned flexible ultralong HAP nanowires was obtained by moving the injecting needle along random directions (Figure 11F). The textiles with different shapes could be easily prepared according to the design. A complex well-designed flexible architecture made from the nanoropes consisting of highly aligned ultralong HAP nanowires was prepared by injecting the solvothermal product slurry into ethanol along predetermined directions according to a designed pattern (Figure 11G) [39].

Surface modification can result in ultralong HAP nanowires with multiple functionalities. For example, if ultralong HAP nanowires were modified with magnetic Fe_3_O_4_ nanoparticles, magnetic nanoropes consisting of highly ordered ultralong HAP nanowires could be obtained [40]. The as-prepared magnetic nanorope could bend reversibly in response to the external magnet field, and the magnetic nonwoven fabric made from magnetic nanoropes could be tightly attracted to a magnet. In addition, the nanoropes could be doped with rare earth ions or hybridized with dyes to exhibit the luminescent function. For instance, when the nanoropes were dyed with rhodamine B and woven into well-designed architectures, these architectures showed a bright red color under UV irradiation [40]. Therefore, this novel kind of ultralong HAP nanowire nanorope may be engineered into fireproof clothing and functional textiles.

### 6.4. Biomedical Applications

Owing to the high biocompatibility and high bioactivity, HAP-based biomaterials have attracted much attention for various biomedical applications, including bone defect repair, dental restoration, wound healing, bio-imaging, and drug delivery [30,31]. However, many traditional HAP-based biomaterials show poor deformability and high brittleness, unless the HAP contents in the composite materials are low. This is because the traditional HAP biomaterials consist of HAP particles, rods, or sheets, and these building units have low aspect ratios and poor flexibility. Therefore, it is challenging to prepare deformable HAP-based biomaterials.

With the merits of high flexibility, ultrasmall diameters, and ultrahigh aspect ratios, ultralong HAP nanowires provide an ideal candidate for the construction of deformable biomaterials. In order to enhance the mechanical properties and biological functions of ultralong HAP nanowire-based biomaterials, efforts have been devoted to assembling them into biomimetic structures. It is well-known that the more similar the materials’ structures are to those of natural materials, the more superior functions the materials can perform. In the previous studies of the authors’ research group, we have successfully developed flexible biomedical paper (bio-paper) [105,106], hydrogels [44], and aerogels [42] based on ultralong HAP nanowires, and they show promising applications in various biomedical fields, for example, bone defect repair [43,79,94,107], wound healing [108,109,110], antibacterial paper [111], and medical test paper [112]. Based on the advances in the studies of ultralong HAP nanowires, the authors’ research group proposed the concept of deformable biomaterials based on ultralong HAP nanowires, and the strategy of using flexible ultralong HAP nanowires to address the high brittleness problem of HAP-based biomaterials [113].

Huang et al. [94] investigated highly porous and elastic aerogel based on ultralong HAP nanowires for high-performance bone regeneration and neovascularization. Importantly, the HAP nanowire aerogel was highly elastic and flexible, thus it can easily fill in bone defects with any shapes especially irregular shapes. The HAP nanowire aerogel could greatly enhance the adhesion, proliferation, and migration of rat bone marrow-derived mesenchymal stem cells, and promote the protein expressions of osteogenesis- and angiogenesis-related genes.

**Figure 12:**
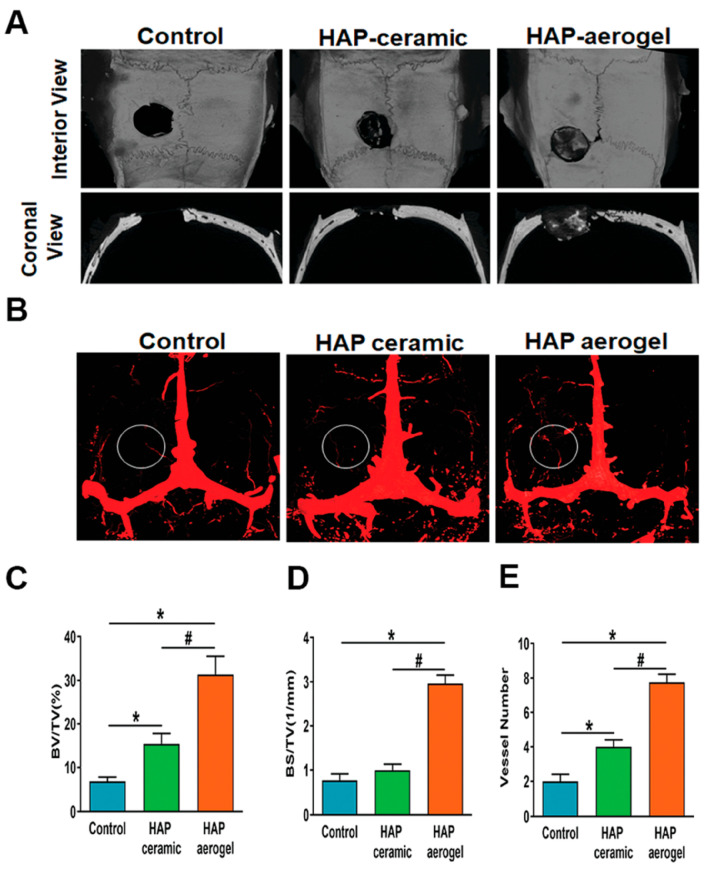
Micro-CT assessment of newly formed bone and blood vessels in rat calvarial defect regions after implantation of the HAP nanowire aerogel scaffold and HAP nanorod ceramic sample for 12 weeks. (**A**) Three-dimensional and coronal views of reconstructed calvarial images. (**B**) Three-dimensional reconstructed images of newly formed blood vessels after Microfil perfusion. The white-circled areas indicate differences in angiogenesis among different groups. (**C**) Bone volume (BV)/total volume (TV), (**D**) bone surface density (BS/TV), and (**E**) blood vessel number in the defects. *, *p* < 0.05 compared to the blank control group; #, *p* < 0.05 compared to the HAP nanorod ceramic sample. Reproduced with permission from ref. [94]. Copyright 2021, The Royal Society of Chemistry.

The animal experiments indicated that the HAP nanowire aerogel scaffold could promote the formation and growth of new bone and neovascularization in the bone defect region. A critical-sized rat calvarial defect model was used to evaluate the performance of the HAP nanowire aerogel scaffold for in vivo bone defect repair (Figure 12). At 12-weeks post-implantation, the HAP nanowire aerogel scaffold showed significant new bone formation in the defect region and the newly formed bone grew deep in the HAP nanowire aerogel scaffold without any obvious crack. In contrast, less new bone formed on the surface of the HAP nanorod ceramic scaffold, and almost no new bone formation was observed in the defect region without any scaffold implantation (Figure 12A). The quantitation of new bone formation indicated that both the ratio of bone volume to total volume (BV/TV) and bone surface density (BS/TV) were significantly higher in the HAP nanowire aerogel group (31.83 ± 7.21% and 2.96 ± 0.33, respectively) compared with the HAP nanorod ceramic group (15.42 ± 4.09% and 0.99 ± 0.23, respectively) and blank control group (6.87 ± 1.61% and 0.77 ± 0.24, respectively) (Figure 12C,D). Furthermore, more new blood vessels were observed in the bone defect region transplanted with the HAP nanowire aerogel scaffold compared with the HAP nanorod ceramic scaffold and the control group, indicating that the HAP nanowire aerogel scaffold showed superior performance for enhancing the angiogenesis in the bone defect region. The blood vessel number of the HAP nanowire aerogel scaffold (7.75 ± 0.96) was significantly larger than those of the HAP nanorod ceramic scaffold (4.00 ± 0.81) and control group (2.00 ± 0.82) (Figure 12B,E). At 12 weeks after the implantation of the scaffolds, the entire bone defect region in the HAP nanowire aerogel scaffold group was filled with a thick bone matrix consisting of abundant fibrous connective tissue and collagen fibers. In contrast, less fibrous connective tissue was observed in the HAP nanorod ceramic and control groups [94].

In a similar work, Sun et al. [43] reported a biomimetic porous scaffold consisting of ultralong HAP nanowires and collagen with 66.7 wt.% HAP nanowires for application in bone defect repair. Compared to the pure collagen as a control sample, the biomimetic porous scaffold consisting of ultralong HAP nanowires and collagen showed greatly enhanced mechanical properties, and the rehydrated biomimetic porous scaffold showed elastic properties. The biomimetic porous scaffold could greatly enhance bone regeneration compared with the pure collagen sample.

Geng et al. [79] prepared the biomimetically ordered ultralong HAP nanowire-based hierarchical hydrogel scaffold with the multi-layered concentric circular structure, osteoimmunomodulatory, and osteogenesis abilities for promoting bone defect repair. Ultralong HAP nanowires were assembled into highly ordered nanowire bundles to form the multi-layered concentric cylinder structure, which was reinforced with hyaluronic acid methacrylate and functionalized with MgAl-layered double hydroxide nanosheets. The well-ordered microstructure and functional component of biomimetic hydrogel scaffold could direct migration and promote the differentiation of bone mesenchymal stem cells and regulate the polarization of macrophages. The in vitro and in vivo experiments indicated that the biomimetic hydrogel scaffold could reduce inflammation, create favorable osteoimmune microenvironment, and promote bone regeneration.

**Figure 13:**
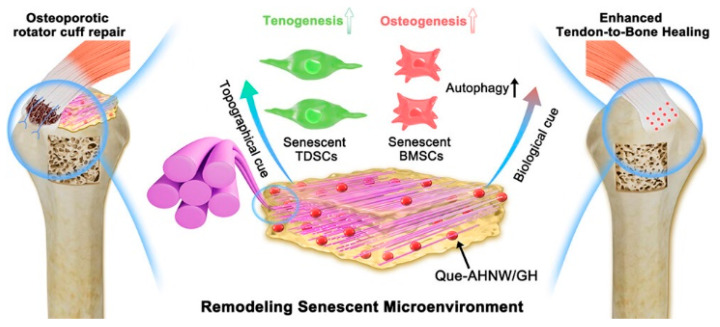
Schematic diagram of the composite hydrogel consisting of quercetin-loaded aligned ultralong HAP nanowires, gelatin, and hyaluronic acid for remodeling the senescent microenvironment through biological and topological routes to promote tendon-to-bone healing in the osteoporotic rotator cuff injury model of rats. Reproduced with permission from ref. [114]. Copyright 2025, American Chemical Society.

Osteoporotic tendon-to-bone healing is currently a significant challenge because cellular senescence disrupts tissue regeneration and weakens repair efficiency. Song et al. [114] reported remodeling the senescent microenvironment for promoting osteoporotic tendon-to-bone healing by synergizing senolytic quercetin and aligned nanowire-structured hydrogels (Figure 13). A composite hydrogel consisting of quercetin-loaded aligned ultralong HAP nanowires, gelatin, and hyaluronic acid was designed and prepared. The composite hydrogel could remodel the senescent microenvironment, reduce senescence in both bone marrow mesenchymal stem cells and tendon-derived stem cells, and promote osteogenesis and tenogenesis. In vivo, the composite hydrogel could enhance bone tunnel regeneration, tendon repair, and tendon-to-bone integration in osteoporotic rats with rotator cuff injury. This composite hydrogel promoted biomechanical strength and gait performance, and it possessed high biosafety.

**Figure 14:**
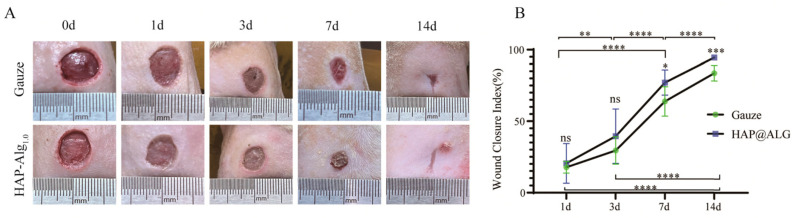
The composite dressing consisting of an ultralong HAP nanowire bio-paper and calcium alginate hydrogel could effectively promote wound healing. (**A**) Representative wound closure images from day 0 to day 14 using the composite dressing or sterile gauze as the control. (**B**) Quantification of wound healing rates at four time points. Asterisks indicate significant *p*-values: * *p* < 0.05; ** *p* < 0.01; *** *p* < 0.001; **** *p* < 0.0001 compared with the control group. ns: non-significant. Reproduced with permission from ref. [110]. Copyright 2025, The Royal Society of Chemistry.

An acute wound is the most common type of skin injury. Developing wound dressings with excellent mechanical and biological properties and angiogenic and therapeutic effects is desirable for wound healing application, yet remains a challenge. Zhu et al. [110] designed and prepared a composite dressing consisting of an ultralong HAP nanowire bio-paper and calcium alginate hydrogel. The HAP bio-paper assembled by ultralong HAP nanowires showed a highly flexible and interwoven structure to enhance the mechanical and protective performance of an alginate hydrogel, and the alginate matrix provided a moist environment for skin regeneration and resistance to swelling and shrinkage, in addition to a reliable bacterial shielding ability. Furthermore, the moisture environment enabled the release of Ca^2+^ ions to promote angiogenesis, accelerate re-epithelialization, and reduce scar formation. In vitro studies reveal that the composite dressing exhibited high biocompatibility and promoted cell migration and angiogenesis and calcium ion influx. Animal in vivo experiments demonstrated that the composite dressing could accelerate wound healing and closure, enhance collagen deposition, and induce neovascularization (Figure 14).

### 6.5. Solar Energy-Driven Seawater Desalination and Wastewater Purification

Amidst the escalating global water crisis driven by water pollution and freshwater scarcity, solar energy-driven water evaporation technology has emerged [115]. It is accomplished by a water evaporator, which absorbs sunlight and directly converts the solar energy to heat for water (seawater or wastewater) evaporation, followed by the collection of condensed clean water from steam [116]. Heat management is a key consideration to enhance the water evaporation efficiency, and it influences the salt deposition behaviors of the evaporator [117]. In the water evaporator, the thermal insulation material is used to localize the heat and thus impede heat loss from the heating zone to bulk water [118].

Thanks to the low thermal conductivity and the easy assembly of ultralong HAP nanowires, they can be engineered into the porous frameworks as the heating insulating components in solar energy-driven water evaporators. Therefore, ultralong HAP nanowires are excellent building blocks for the construction of high-performance solar energy-driven water evaporators.

In order to reduce the water transportation resistance for highly efficient evaporation, the ultralong HAP nanowire-based evaporators have been engineered with a tree-/lotus stem-inspired vertically aligned channel structure or a fish gill-inspired multi-scale-ordered structure [80,84,86]. A common feature of these structures is the ordered channels, which allow for rapid water transportation, fast steam escape, and high water evaporation rate. The ultralong HAP nanowire-based evaporators exhibited high solar energy absorption and photothermal conversion efficiency. Moreover, the ordered channel structure was able to accelerate the ion diffusion and salt–water exchange and prevent the solid salt deposition on the surface of evaporators. As a result, the ultralong HAP nanowire-based evaporators showed high water evaporation rates, suggesting their potential application in sustainable, highly efficient, salt-resistant seawater desalination and wastewater purification to produce clean fresh water.

**Figure 15:**
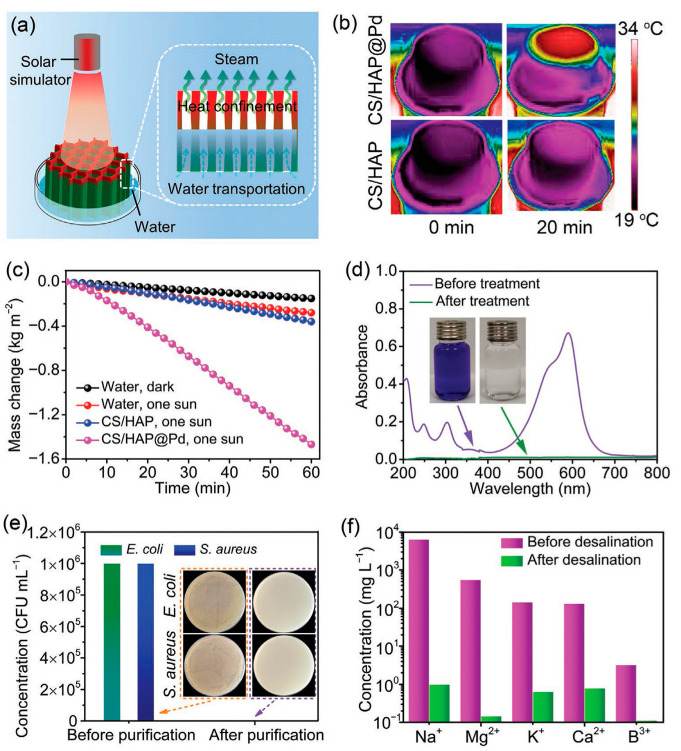
Performances of the tree-inspired CS/HAP@Pd biomimetic aerogel with vertically aligned channels consisting of ultralong HAP nanowires, Pd nanoparticles, and chitosan for solar energy-driven water purification and seawater desalination. (**a**) Schematic illustration of solar energy-driven steam generation based on the tree-inspired CS/HAP@Pd biomimetic aerogel. (**b**) Infrared (IR) thermal images of the CS/HAP@Pd aerogel and the CS/HAP aerogel without and under one sun illumination for 20 min. (**c**) Weight changes in pure water versus solar light irradiation time under one sun illumination: pure water in the absence of any sample in darkness or under one sun illumination, pure water with the CS/HAP@Pd aerogel, or the CS/HAP aerogel under one sun illumination. (**d**) UV–vis absorption spectra and the corresponding digital images of the aqueous solution containing crystal violet (CV), Congo red (CR), and NaBH_4_ before treatment and the collected condensed water after solar energy-driven purification. (**e**) Bacterial concentrations of the aqueous suspension containing *E. coli* or *S. aureus* before and after solar energy-driven purification; digital images of the solid nutrient agar plates cultured with the aqueous suspension containing *E. coli* or *S. aureus* (left) and the condensed water after solar energy-driven purification (right). (**f**) Concentrations of five main ions in the actual seawater sample and the condensed clean water after solar energy-driven desalination. Reproduced with permission from ref. [84]. Copyright 2021, Wiley-VCH.

As a typical example, inspired by water transportation and transpiration of natural trees, Xiong et al. [84] prepared an ultralong HAP nanowire-based biomimetic aerogel with vertically aligned channels by unidirectional freeze-drying for solar energy-driven water purification and seawater desalination (Figure 15). Ultralong HAP nanowires acted as the carrier to immobilize and stabilize Pd nanoparticles as the nanocatalyst. Pd nanoparticle-modified ultralong HAP nanowires were prepared by the combination of the calcium oleate precursor solvothermal method and in situ reduction process. Then, the aqueous dispersion containing Pd nanoparticle-modified ultralong HAP nanowires, chitosan, and a cross-linking agent was freeze-cast and vacuum-dried. After thermal curing treatment, a tree-inspired CS/HAP@Pd biomimetic aerogel with vertically aligned channels was obtained. Owing to the tree-analogical structure and functions, the biomimetic aerogel showed vertically aligned channels, hydrophilicity, mechanical robustness, high water flux, excellent anti-biofouling, and superior photothermal conversion ability, enabling high performance in gravity-driven continuous flow catalysis and disinfection and solar energy-driven steam generation, water purification, and seawater desalination (Figure 15a). The CS/HAP@Pd aerogel showed superior photothermal conversion and heat confinement properties; the surface temperature of the CS/HAP@Pd aerogel increased gradually and reached 30.9 °C under one sun irradiation (1 kW m^–2^) for 20 min (Figure 15b). The water evaporation rate was 1.47 kg m^−2^ h^−1^ under one sun irradiation in the presence of the CS/HAP@Pd aerogel (Figure 15c). In addition, no salt crystal deposition was observed on the top surface of the CS/HAP@Pd aerogel after 10 h of continuous solar desalination. Furthermore, a simulated industrial dye wastewater sample was prepared using crystal violet (CV), Congo red (CR), and NaBH_4_; both the collected condensed water and residual bulk solution after solar energy-driven purification and catalytic degradation were colorless and transparent, and the absorption peaks of CV and CR disappeared in the UV–vis spectra of two samples (Figure 15d). The dye molecules in the bulk water could be efficiently degraded through the catalytic reaction, resulting in contaminant-free clean water. In the anti-bacteria experiments, the aqueous solution containing Escherichia coli (E. coli) or Staphylococcus aureus (S. aureus) with a concentration of 1 × 10^6^ CFU mL^−1^ was used as the simulated microbial wastewater sample. After purification using the CS/HAP@Pd aerogel under solar light irradiation, the concentration of bacteria in the collected condensed water was zero, and no bacterial colonies were found on the solid nutrient agar plate (Figure 15e). Moreover, an actual seawater sample obtained from the East China Sea was used for testing the solar energy-driven seawater desalination performance, and the concentrations of five main ions (Na^+^, Mg^2+^, K^+^, Ca^2+^, and B^3+^) decreased to <1 mg L^−1^ after solar energy-driven seawater desalination using the CS/HAP@Pd aerogel (Figure 15f); the collected clean water could meet the drinking water standards as defined by the World Health Organization and U. S. Environmental Protection Agency [84].

### 6.6. Applications in Energy Fields

Owing to their high flexibility and thermal stability, ultralong HAP nanowires are promising for applications in various energy fields. The previous studies of the authors’ research group demonstrate the feasibility and superiority of ultralong HAP nanowires for applications in batteries and capacitors, for example, fire-resistant separators in advanced lithium-ion batteries [119] and fire-resistant electrodes and separators in lithium-ion capacitors [120].

Solid-state polymer electrolytes are important for advancing solid-state lithium metal batteries. However, their wide-spread applications are hindered by low ionic conductivity at room temperature and lithium dendrite growth. Importantly, the biomimetic materials assembled with ultralong HAP nanowires can facilitate the enhancement of properties of batteries. As a typical example, inspired by the vertically aligned channel structure of natural trees, Xie et al. [121] designed and prepared the highly ionic conductive composite membrane electrolyte with a vertically aligned structure and radial gradient copolymer for high-performance solid-state lithium metal batteries. They designed and constructed a novel solid-state composite membrane electrolyte combining the vertically aligned channel structure and the copolymer with a radial gradient composition. Within the vertically aligned channels, the composition of poly(vinyl ethylene carbonate-co-poly(ethylene glycol) diacrylate) changed in a gradient along the radial direction: from the center to the wall of vertically aligned channels, the proportion of vinyl ethylene carbonate in the copolymer decreased, while the proportion of poly(ethylene glycol) diacrylate increased accordingly. The as-prepared solid-state composite membrane electrolyte exhibited a high critical current density of 1.2 mA cm^−2^ and high ionic conductivity of 2.03 mS cm^−1^ at room temperature. By adopting this composite membrane electrolyte, a Li//Li symmetric cell showed a stable cycling performance for over 1850 h at 0.2 mA cm^−2^/0.2 mA h cm^−2^, and 77.3% capacity retention was achieved for a Li//LiFePO_4_ battery at 2 C after 300 cycles.

## 7. Conclusions

Ultralong HAP nanowires are a novel kind of nonmetal oxide flexible nanofibers with rich hydroxyl groups, diameters smaller than 100 nm (typically ~10 nm), lengths of hundreds of micrometers, and ultrahigh aspect ratios more than 10,000. Owing to these merits, ultralong HAP nanowires show high flexibility and excellent biocompatibility, and they can act as flexible nano-scale building blocks to construct macroscopic biomimetic materials with both high strength and high toughness, which can solve the high brittleness problem of traditional HAP materials.

When considering synthetic strategies, factors such as the cost, yield, environmental friendliness, and time consumption must be considered. Ultralong HAP nanowires can be synthesized by the calcium oleate precursor hydrothermal/solvothermal method (using various alcohols as the solvent for the solvothermal method, and water as the only solvent for the hydrothermal method, as well as different inorganic phosphate salts or some phosphorus-containing biomolecules (such as ATP and creatine phosphate) as the phosphorus sources). In the case of organic phosphorus-containing biomolecules as the phosphorus sources, the chemical reaction, crystal nucleation, and growth processes can be regulated by the hydrolysis conditions of phosphorus-containing biomolecules. In addition, the preparation time can be significantly shortened from tens of hours required by traditional heating to less than one hour by combining rapid microwave heating with the calcium oleate precursor hydrothermal/solvothermal method. The large-scale and low-cost production of flexible ultralong HAP nanowires is a prerequisite for their practical applications. Currently, the large-scale production of ultralong HAP nanowires has been realized in the authors’ laboratory using a stainless steel autoclave with a volume of 100 L. We believe that the industry-scale production of ultralong HAP nanowires may be realized in the future.

Inorganic nanofibers should have long lengths, small diameters, and high aspect ratios to achieve high flexibility. This increases the difficulty in the formation of the ordered assembly of inorganic nanofibers because they tend to intertwine with each other. Therefore, the preparation of the liquid crystal slurry such as the ultralong HAP nanowire liquid crystal slurry is desirable. In the liquid crystal slurry, the quasi-long-range orderly arrangement of flexible ultralong HAP nanowires can be further assembled into macroscopic ordered structures with the assistance of external forces and fields, and it can also be printed into well-designed structural materials with complex shapes and sizes by 3D printing technology. The key to realizing the formation of flexible inorganic nanofiber liquid crystals lies in using suitable regulators that can induce mutual attractions among inorganic nanofibers, leading to their ordered arrangement at the micro-scale, while ensuring the quasi-assemblies remain stably dispersed within the slurry.

As discussed above, inspired by various natural materials, different biomimetic structures assembled with flexible ultralong HAP nanowires can be designed and constructed to prepare diverse high-performance biomimetic materials for applications across multiple fields. These successful achievements provide valuable insights into the design, fabrication, and application of high-performance biomimetic materials based on other flexible inorganic nanofibers. The assembled structures of flexible inorganic nanofibers significantly influence the properties and macroscopic functionalities of biomimetic materials. Biomineralized tissues in organisms have optimized their structures and compositions through a long evolutionary process, providing us with optimal structural design and assembly strategies. Therefore, the selection of structural models can refer to those of natural materials for designing high-performance biomimetic materials. For example, most reported biomimetic materials consisting of ultralong HAP nanowires show hierarchically ordered structures resembling those of biomineralized tissues from living organisms, such as dental enamel, compact bone, cancellous bone, and nacre. Other structure models are from natural organic fibrous materials in plants, such as trees and plant stems, and their design principles can help prepare advanced functional materials with high mechanical properties and tailored porous/channel structures. Furthermore, we have demonstrated the successful design and construction of novel biomimetic materials with superior properties and multiple functions consisting of flexible ultralong HAP nanowires based on the combination of multiple biomimetic structural models.

Based on the guidelines derived from the structure-and-property relationships of natural materials, ultralong HAP nanowires can be assembled into a variety of biomimetic materials with hierarchically ordered structures and multiple functionalities. In this paper, we have comprehensively discussed the reported bioinspired materials based on ultralong HAP nanowires. The enamel-inspired materials with multi-scale ordered structures show significant mechanical enhancement. When ultralong HAP nanowires are assembled into the nacre-mimetic “brick-and-mortar” structure, strong and tough biomimetic composite materials can be obtained. By integrating the structural features of both enamel and nacre, a novel bioinspired “fiberboard-and-mortar” ordered structure with a combination of high strength and high toughness was developed. The mechanical strength of the biomimetic materials can be significantly enhanced by assembling ultralong HAP nanowires into the hierarchically ordered structure that mimics the mineralized collagen fibril array of compact bone. The cancellous bone-like ultralong HAP nanowire aerogel possesses ultrahigh porosity and excellent elasticity. The ultralong HAP nanowire-based porous scaffold resembling the multi-layer concentric circular structure of bone can provide a favorable osteogenic and immune microenvironment for bone regeneration. In addition, the ultralong HAP nanowire-based solar water evaporators with tree-/lotus stem-inspired vertically aligned channel structures or a fish gill-inspired multi-scale-ordered structure can facilitate water transportation, enhance the water evaporation rate, and inhibit salt deposition. The superior performances of ultralong HAP nanowire-based biomimetic materials make them highly competitive in applications of mechanical protection, heat insulation, flexible devices, biomedicine and healthcare, and solar energy-driven seawater desalination and wastewater purification, greatly extending the application scopes of HAP-based materials from traditional ceramics to the fields of novel flexible multifunctional materials.

Despite many merits and exciting research developments of ultralong HAP nanowires and their derived high-performance biomimetic materials, there are also some limitations that should be considered. From a chemical stability perspective, although ultralong HAP nanowires are highly stable under alkaline conditions, they are not acid-resistant; thus, ultralong HAP nanowires are unstable and gradually dissolve under acidic conditions. Fortunately, another kind of flexible inorganic nanofiber, BaSO_4_ nanofibers, has been developed by the authors’ research group, which can address this limitation, because BaSO_4_ nanofibers exhibit high stability under both acidic and alkaline conditions.

## 8. Future Outlook

Owing to the advantages of flexible inorganic nanofibers, it is expected that the research work on flexible inorganic nanofibers, especially ultralong HAP nanowires, will continuously intensify in coming years, and as a result, more and more high-performance biomimetic materials based on flexible inorganic nanofibers including ultralong HAP nanowires will emerge in the future. Although exciting advances and developments have been achieved, there are still some important scientific issues that need to be addressed. The proposed main future research directions in this exciting and rapidly evolving research field are as follows: (1) in order to realize the practical applications, low-cost and eco-friendly synthetic methods need to be developed for the large-scale production of flexible inorganic nanofibers including ultralong HAP nanowires; (2) by mimicking the intracrystalline structure (such as intracrystalline inclusion, ion substitution, and amorphous phase) of natural biominerals, the properties of biomimetic materials based on flexible inorganic nanofibers including ultralong HAP nanowires can be enhanced through bioinspired intracrystalline engineering; (3) high-performance advanced functional biomimetic materials with novel complex structures can be obtained by mimicking the structural models of biomineralized tissues from living organisms, such as dental enamel, compact bone, cancellous bone, and nacre, and by mimicking structure models from natural organic fibrous materials in plants, such as trees and plant stems, which can help prepare advanced functional materials with high mechanical properties and tailored porous/channel structures; (4) novel biomimetic materials with superior properties and multiple functions can be designed and constructed by the combination of multiple biomimetic structural models using flexible inorganic nanofibers including ultralong HAP nanowires; (5) multiple kinds of flexible inorganic nanofibers can be employed as cooperative nano-scale building blocks to construct functional biomimetic materials with superior comprehensive properties; (6) by designing the bioinspired assembly structures of flexible inorganic nanofibers including ultralong HAP nanowires, intelligent biomimetic materials can be fabricated, which not only exhibit high mechanical properties, but also integrate multiple functions such as sensing, self-healing, and information processing; (7) with advances in artificial intelligence, AI-assisted designs and fabrication are promising to accelerate the development of flexible inorganic nanofibers including ultralong HAP nanowires and their derived high-performance biomimetic materials with superior customized structures and functions; and (8) investigating various properties and exploring the applications in a variety of fields of the biomimetic materials constructed with flexible inorganic nanofibers including ultralong HAP nanowires.

## Data Availability

No new data were created or analyzed in this study; additional information will be shared upon reasonable request to the corresponding author.
